# Recurring volcanic winters during the latest Cretaceous: Sulfur and fluorine budgets of Deccan Traps lavas

**DOI:** 10.1126/sciadv.adg8284

**Published:** 2023-10-04

**Authors:** Sara Callegaro, Don R. Baker, Paul R. Renne, Leone Melluso, Kalotina Geraki, Martin J. Whitehouse, Angelo De Min, Andrea Marzoli

**Affiliations:** ^1^ Centre for Earth Evolution and Dynamics, University of Oslo, Sem Sælands vei 2A, 0371 Oslo, Norway.; ^2^ Centre for Planetary Habitability, University of Oslo, Sem Sælands vei 2A, 0371 Oslo, Norway.; ^3^ Department of Earth and Planetary Sciences, McGill University, 3450 University St., Montreal, QC, Canada.; ^4^ Berkeley Geochronology Center, 2455 Ridge Road, Berkeley, CA 94709, USA.; ^5^ Department of Earth and Planetary Science, University of California, Berkeley, Berkeley, CA 94720, USA.; ^6^ Dipartimento di Scienze della Terra, Ambiente e Risorse, University of Napoli Federico II, via Cintia 21, 80126 Napoli, Italy.; ^7^ Diamond Light Source, Harwell Science and Innovation Campus, Didcot OX11 0DE, UK.; ^8^ Swedish Museum of Natural History, Stockholm, Sweden.; ^9^ Department of Mathematics and Geoscience, University of Trieste, via Weiss 2, 34128 Trieste, Italy.; ^10^ Dipartimento Territorio e Sistemi Agro-Forestali, University of Padova, 35020 Legnaro, Italy.

## Abstract

Two events share the stage as main drivers of the Cretaceous-Paleogene mass extinction—Deccan Traps volcanism, and an asteroid impact recorded by the Chicxulub crater. We contribute to refining knowledge of the volcanic stressor by providing sulfur and fluorine budgets of Deccan lavas from the Western Ghats (India), which straddle the Cretaceous-Paleogene boundary. Volcanic fluorine budgets were variable (400 to 3000 parts per million) and probably sufficient to affect the environment, albeit only regionally. The highest sulfur budgets (up to 1800 parts per million) are recorded in Deccan lavas emplaced just prior (within 0.1 million years) to the extinction interval, whereas later basalts are generally sulfur-poor (up to 750 parts per million). Independent evidence suggests the Deccan flood basalts erupted in high-flux pulses. Our data suggest that volcanic sulfur degassing from such activity could have caused repeated short-lived global drops in temperature, stressing the ecosystems long before the bolide impact delivered its final blow at the end of the Cretaceous.

## Teaser

Late Cretaceous Deccan basalts were sulfur- and fluorine-rich. Degassing potentially caused cold spells and ecosystem damage.

## INTRODUCTION

**Fig. 1.**
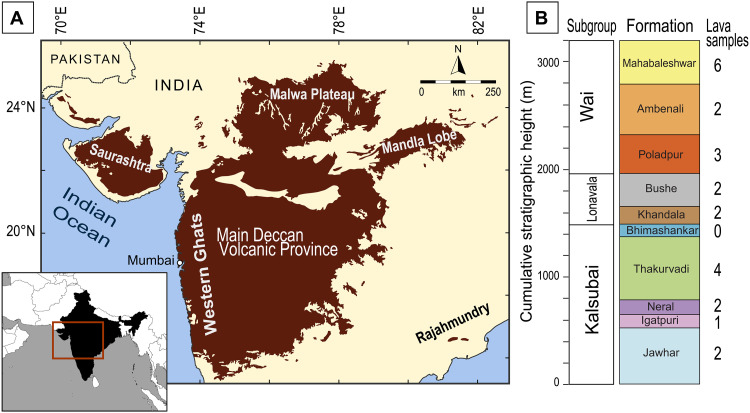
Deccan Traps large igneous province overview. (**A**) Present-day distribution of the Deccan Traps in India. Samples investigated in this study come from the WG Escarpment, where the thickest and most complete lava piles are preserved. (**B**) Schematic volcanostratigraphy for the Main Deccan Volcanic Province (MDVP) in the WG (*6*). Ten lava formations and three subgroups are reported along with the number of samples analyzed for each formation. In this list, and in the dataset, we include three samples (D231, D241, and D244) collected at Mahabaleshwar that were previously analyzed by our group (*31*).

**Fig. 2.**
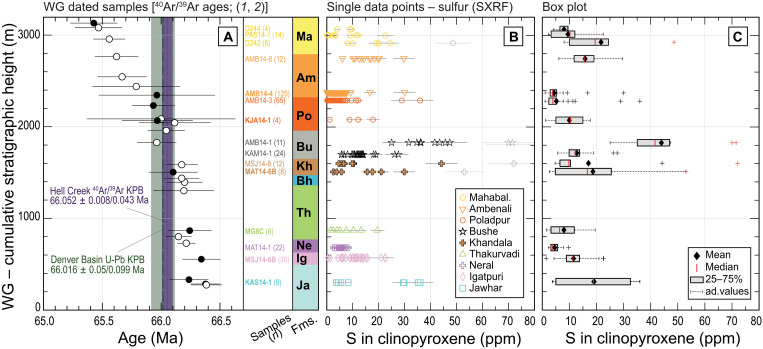
Sulfur concentrations (in parts per million) measured in clinopyroxenes from the Western Ghats lava pile. (**A**) Investigated lava samples plotted in stratigraphic order, *y* axis in meters of cumulative stratigraphic height. Six of the analyzed samples were previously dated by high-precision ^40^Ar/^39^Ar geochronology (*1*, *2*) and are labeled in bold, with ages plotted as black circles. Additional ^40^Ar/^39^Ar ages from other samples from the same lava pile are plotted as white circles. Uncertainty on each ^40^Ar/^39^Ar age is shown at ±2 SD, including systematic sources. Age ranges for the KPB are reported in dotted purple for the ^40^Ar/^39^Ar dataset (66.052 ± 0.008/0.04 Ma; external uncertainty in purple shade) (*1*, *2*) and in stapled green for the U-Pb dataset (66.016 ± 0.05/0.099 Ma; external uncertainty in green shade) (*3*, *41*). Lava formations: Ja, Jawhar; Ig, Igatpuri; Ne, Neral; Th, Thakurvadi; Bh, Bhimashankar; Kh, Khandala; Bu, Bushe; Po, Poladpur; Am, Ambenali; Ma, Mahabaleshwar. (**B**) Sulfur concentrations measured by synchrotron light x-ray fluorescence (SXRF) (*n* = 353 data points) in clinopyroxenes from 15 lava samples. Individual data points with their associated error bars (1 SD) are plotted. (**C**) Sulfur concentrations in clinopyroxene are reported as box plots, highlighting the petrological and statistical outliers. Each box extends from the 25th to the 75th percentile of the sample analyses, with the median reported as a red line and the mean as a black diamond. The whiskers extend outside each box from the end of the interquartile range to the furthest concentration within the whisker length (the adjacent value). Measured concentrations beyond the whisker length (i.e., more than 1.5 times the interquartile range) are marked as outliers, with crosses. Black crosses mark statistical outliers. Red crosses mark petrological outliers. The latter are reported in red in data S3 and not plotted in Fig. 4B.

**Fig. 3.**
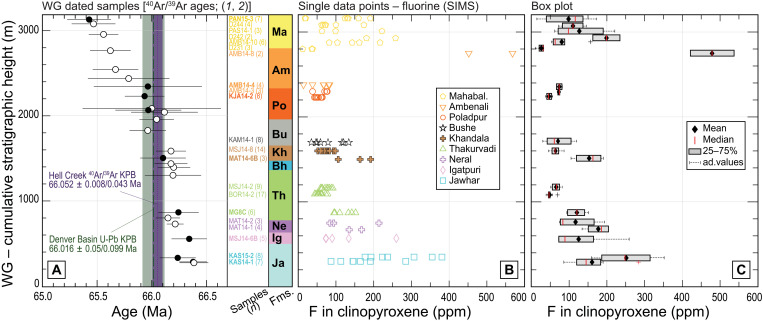
Fluorine concentrations (in parts per million) measured in clinopyroxenes from the Western Ghats lava pile. (**A**) Investigated lava samples plotted in stratigraphic order, *y* axis in meters of cumulative stratigraphic height. Eight of the analyzed samples were previously dated by high-precision ^40^Ar/^39^Ar geochronology (*1*, *2*) and are labeled in bold, with ages plotted as black circles. Additional ^40^Ar/^39^Ar ages from other samples from the same lava pile are plotted as white circles. Uncertainty on each ^40^Ar/^39^Ar age ± 2 SD, including systematic sources. Age ranges for the KPB are reported in dotted purple for the ^40^Ar/^39^Ar dataset (66.052 ± 0.008/0.04 Ma; external uncertainty in purple shade) (*1*, *2*) and in stapled green for the U-Pb dataset (66.016 ± 0.05/0.099 Ma; external uncertainty in green shade) (*3*, *41*). (**B**) Fluorine concentrations measured by secondary ion mass spectrometry (SIMS) (*n* = 126 data points) in clinopyroxenes from 21 lava samples, plotted as individual data points. Associated error bars (±1 SD) are smaller than the symbol. (**C**) Fluorine concentrations measured in clinopyroxene are reported as box plots. Details on the box plot construction as in Fig. 2.

**Fig. 4.**
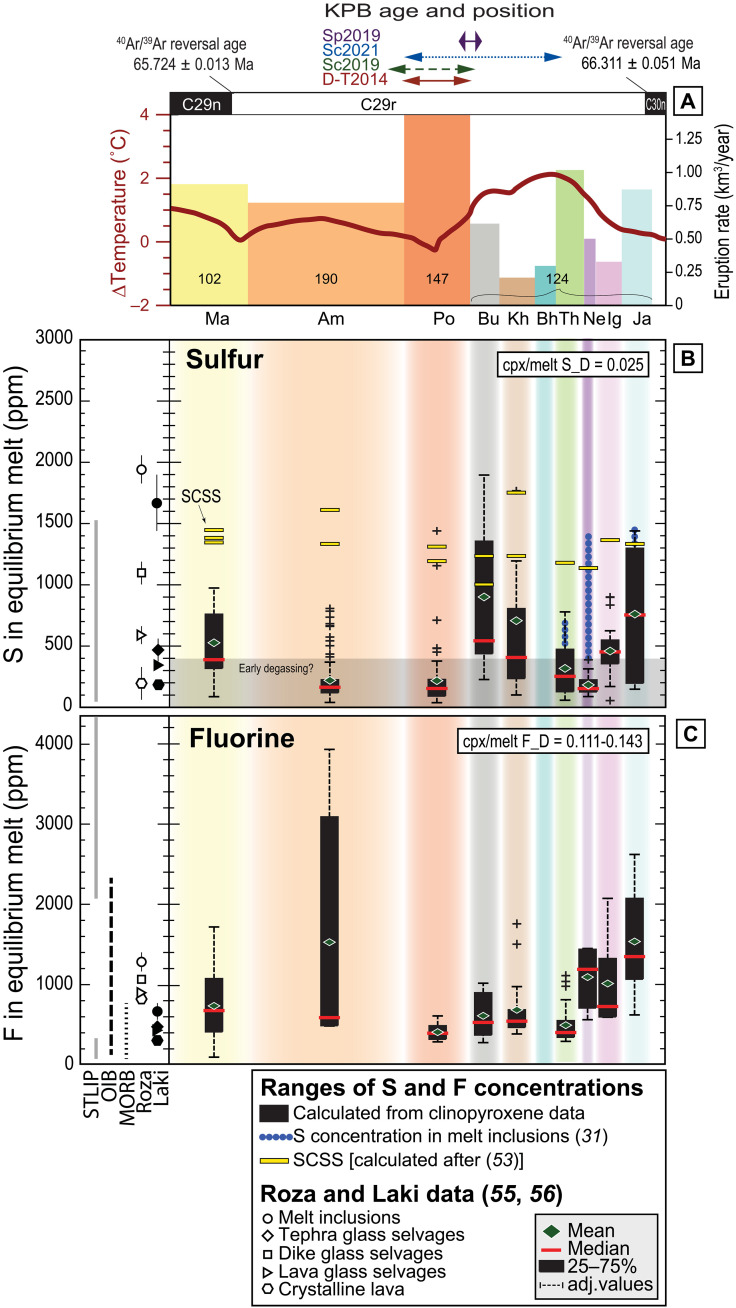
S and F concentrations calculated for Deccan Traps melts in equilibrium with the analyzed clinopyroxenes. (**A**) Eruption rates for the WG Formations of the MDVP (*1*, *41*) based on approximate duration (horizontal length) and eruptive volume calculated by Richards *et al*. (*36*). The volume of each formation is 10^3^ km^3^. Cumulative volumes reported for Kalsubai and Lonavala Subgroups (details in data S4). Red curve: Global temperature change across the KPB as 60-point fast Fourier transform smoother through a compilation of benthic δ^18^O [‰ Vienna Pee Dee Belemnite (VPDB)] data (*14*). Curve plotted correlating magnetic reversals from (*1*, *14*). Purple arrow: ^40^Ar/^39^Ar KPB age (66.016 ± 0.08/0.004 Ma) and highest probability stratigraphic position along WG (*1*, *2*, *41*). Blue dotted arrow: Probability of stratigraphic position of KPB age from (*1*, *2*) according to (*41*). Green stapled arrow: U-Pb KPB age (66.016 ± 0.05/0.099 Ma) and its highest probability position along the WG (*3*, *24*, *41*). Red arrow: Astronomically calibrated KPB age (66.022 ± 0.040 Ma) (*87*), tied to the paleotemperature curve of (*14*). Magnetozones from (*78*). ^40^Ar/^39^Ar ages for magnetic reversals from (*1*). (**B** and **C**) Concentration of S (in parts per million) and F (in parts per million) in equilibrium melts calculated from clinopyroxene microanalyses (data S2). Concentrations are plotted per lava formation. Box-and-whiskers construction as in Fig. 2. Yellow horizontal bars in (B): Sulfur concentration at sulfide saturation (SCSS) calculated for each lava sample (*46*). SCSS values of different samples are plotted as stacked and centered on the formation of pertinence. We plotted (B) previously published S concentrations in melt inclusions in olivine and plagioclase from the MDVP (*28*), and S and F concentrations for Laki (Iceland), the Roza member of Columbia River Basalts (USA), and the Siberian Traps (*20*, *48*–*50*) for comparison. We plotted (C) fluorine concentrations measured in oceanic island (OIB) and mid-ocean ridge basalts (MORB) (*61*).

Substantial environmental changes starting in the Late Cretaceous and culminating at the boundary between Cretaceous and Paleogene [KPB; ~66 million years (Ma); (*1*–*3*)] triggered one of the most notorious biotic crises in Earth history, leading to the demise of non-avian dinosaurs and to extinction of up to 60% marine genera (*4*, *5*). This period of intense global transformation was contemporaneous with two large-scale events, each potentially capable of driving such changes—extremely voluminous volcanic activity across the KPB, forming the ca. 10^6^ km^3^ Deccan Traps large igneous province (LIP), located in present-day India and Seychelles (Fig. 1) (*6*, *7*); and a major meteorite impact, as testified by the Chicxulub crater (Mexico) and by a worldwide Ir anomaly (*8*, *9*). Deconvolution of the effects of one trigger from the other is challenging and remains an ongoing, intense debate in the Earth science community (*9*–*16*). Recently, high-precision ^40^Ar/^39^Ar (*1*, *2*) and U-Pb (*3*) age data for the Western Ghats (WG; western India) set the events on a tightly constrained timeline. The emplacement of the Deccan Traps basalts unquestionably straddled the KPB, with voluminous, pulsed eruptions preceding the boundary by 0.3 Ma to 0.4 Ma (lavas of the Kalsubai and Lonavala Subgroups) (Figs. 1 to 4) and continuing for ca. 0.7 Ma after that (Wai Subgroup lava flows) (*1*, *2*). Warming and cooling trends preceding the KPB (*14*, *15*) are interpreted as a signal that degassing from Deccan Traps volcanism was already forcing the global end-Cretaceous (Maastrichtian) climate, possibly enhanced by orbitally driven insolation variations (*16*), and that the Chicxulub impact wreaked further havoc on previously volcanically perturbed global ecosystems (*12*, *17*). Some have argued that Deccan volcanism alone was sufficient to induce a mass extinction and that the Chicxulub impact only served to exacerbate its consequences on the ecosystems (*19*). Conversely, others suggested that the greenhouse forcing of Deccan Traps degassing played a mitigating role on the impact-derived, global winter (*13*, *14*).

Flood basalt volcanism can induce global climate and biotic disruption due to volcanic outgassing (CO_2_, SO_2_, and halogens) and ash production, although the precise mechanisms are only qualitatively constrained (*20*). Recent atmospheric models indicate that the combined volcanic release of sulfur and halogens can amplify atmospheric stress (*21*), highlighting the importance of quantifying multiple-species volatile budgets from past eruptions. The effect of Deccan Traps SO_2_ and CO_2_ emissions has been modeled before (*14*, *22*, *23*), but large uncertainties persist due to the paucity of data on magmatic gas concentrations and on emission rates. In addition, some volatile species may be derived from sedimentary host rocks heated by intruded magmas, and gas discharge may also precede eruption (passive degassing) (*23*, *24*). Quantifying these inputs and temporally constraining them remains a challenge.

Volatile discharge and emplacement rates have been calculated for the Deccan Traps based on terrestrial mercury records and geochemical box models (*25*–*27*) but chemical data obtained directly from the volcanic material are still sparse. Available data on SO_2_ and Cl (*28*) and CO_2_ (*23*, *29*) from the Deccan Traps are mostly derived from melt inclusions found in rare occurrences (16 samples in total) of fresh olivine or plagioclase; estimates of fluorine concentrations have never been reported (*20*, *30*). Furthermore, previously published volatile estimates for the Deccan lavas come from flows that do not span the extinction interval (*1*–*3*), being restricted either to the oldest part of the WG [Kalsubai Subgroup (*20*, *23*, *28*)] or to the youngest basalts [Wai Subgroup (*29*, *31*)], which postdate the KPB. Here, we provide an age-constrained dataset for magmatic concentrations of sulfur and fluorine in 23 Deccan Traps lavas from nine volcanic formations of the WG lava pile straddling the KPB, based on in situ microbeam analyses of sulfur and fluorine in clinopyroxene crystals. We use these analyses to estimate magmatic budgets for these two volatiles by applying available clinopyroxene-liquid partition coefficients (*31*–*33*) and discuss the potential role of volcanic degassing on the climate and biotic crisis marking the transition from the Cretaceous to the Paleogene.

## RESULTS

### Western Ghats volcanostratigraphy and sample set

Straddling the Cretaceous-Paleogene boundary (KPB), at ~66 Ma, about 10^6^ km^3^ of tholeiitic flood basalts was emplaced in present-day India and constitutes the Main Deccan Volcanic Province (MDVP) (*1*–*3*, *6*, *7*, *34*–*36*). Some Deccan lavas can be traced over long distances, up to ~1000 km, and lava geochemistry has been used to stratigraphically correlate and classify flows, starting from the volcanostratigraphy defined on the WG sections, where the lava sequence is thickest (~3.5 km cumulative) and best exposed (*6*, *34*, *37*). Other areas of the Deccan Traps comprise different magma types, including alkaline rocks and carbonatites, that are not comagmatic with the rest of the MDVP [see, e.g., (*38*, *39*)].

The 23 basalts targeted for this study were sampled from 9 of the 12 lava formations of the WG Escarpment lava pile in the MDVP. This composite lava pile includes 12 geochemical formations, divided into three subgroups (Fig. 1B). From the bottom, the Kalsubai Subgroup encompasses the heterogeneous (Mg-rich to evolved) tholeiitic basalt lavas of the Jawhar, Igatpuri, Neral, Thakurvadi, and Bhimashankar Formations. The Khandala and Bushe Formations compose the middle Lonavala Subgroup, with Bushe lavas showing substantial evidence of crustal contamination (*6*, *7*, *34*, *40*). At the top, the Wai Subgroup contains the Poladpur, Ambenali, Mahabaleshwar, Panhala, and Desur Formations, with more voluminous flows displaying dominantly mantle-like geochemical characteristics. High-precision ^40^Ar/^39^Ar data on plagioclase separates from the lavas investigated in our study (Figs. 2A and 3A) (*1*, *2*) constrain the most likely position of the KPB (66.052 ± 0.008/0.04 Ma) between the Bushe and Poladpur lava formations, at the transition between the Lonavala and Wai Subgroups (Fig. 4A). High-precision U-Pb geochronology on zircon from paleosols occurring between lava flows (“red boles” horizons) instead places the KPB (66.016 ± 0.05/0.099 Ma) at slightly higher level in the WG lava pile, within or at the top of the Poladpur Formation (Fig. 4A) (*3*, *24*). Nevertheless, the ^40^Ar/^39^Ar and U-Pb ages for the KPB overlap within uncertainty (Figs. 2A and 3A) (*41*).

The WG lava pile is the best candidate for this study because it is very well characterized in terms of geochemistry and age (*1*–*3*, *6*, *7*, *34*–*37*, *42*). Several of the targeted rocks were dated by the ^40^Ar/^39^Ar method (Figs. 2A and 3A) (*1*, *2*). The stratigraphic position of the studied samples in the various lava formations was identified on the basis of field observations coupled with whole-rock major and trace elements, following previously proposed geochemical classifications (*1*, *6*, *42*). The investigated lavas are low- and high-TiO_2_ tholeiitic basalts and basaltic andesites, chemically representative of their respective formations (fig. S1 and data S1). Most samples have porphyritic and glomeroporphyritic textures, with plagioclase, altered olivine, and clinopyroxene, in a glassy to microcrystalline matrix with feldspar, augite, pigeonite, Fe-Ti oxides, rare sulfides, and native copper (fig. S2 and data S1). The clinopyroxene crystals analyzed for this study are fresh augite phenocrysts, with compositions typical of Ca-rich clinopyroxene in tholeiitic basalts worldwide, including the Deccan Traps (fig. S3A and data S2) [see, e.g., (*43*, *44*)].

Most phenocrysts are in chemical and textural equilibrium with their host lava; we also targeted clinopyroxenes from glomeroporphyritic clots (figs. S2 to S7). Sample BOR14-2 is the only coarse-grained lava, possibly the crystalline interior of a thick lava flow. All the lava samples are sulfide-poor or free, with a minority (7 of 23) presenting small sulfides (maximum of 15 μm) either in the glassy or microcrystalline mesostasis or in secondary fractures (fig. S7).

### In situ determination of sulfur and fluorine in clinopyroxene

We analyzed sulfur by synchrotron light x-ray fluorescence (SXRF) (*31*, *32*) in 28 clinopyroxene phenocrysts of the MDVP, from 13 basalts sampled in the WG lava pile (data S3). Our group previously investigated three clinopyroxenes from two additional lava samples (D242 and D244; Mahabaleshwar Formation) (*31*), and we include those results in this dataset.

Sulfur in clinopyroxenes ranges from <1 part per million (ppm) (the SXRF detection limit) to a maximum of 72 ppm (Fig. 2). We found the highest S concentrations in clinopyroxenes from the Khandala and Bushe lavas of the Lonavala Subgroup (up to 72 ppm). Another relative peak in S concentration is recorded for clinopyroxenes of the Jawhar Formation (up to 36 ppm), while most data from the rest of the crystals show low S (generally <30 ppm).

We generally note a narrow range of S concentrations within the same crystal, except outliers, without substantial variations in S concentration from core to rim, as exemplified by line analyses of up to 48 spots obtained from clinopyroxenes (figs. S4 to S6). However, some crystals show slight sulfur zoning (fig. S4E) or variability in sulfur (figs. S4G to S6C). For some lava samples, we analyzed multiple crystals and found mostly uniform compositions. Sample KAS14-1 is an exception, with bimodal S concentrations (one pyroxene contains 4- to 8-ppm S, whereas another has 30 to 36 ppm). Clinopyroxenes from lavas of the same formation can have different concentrations in S (intraformational variability; e.g., 6- to 30-ppm S measured in crystals from Ambenali sample AMB14-8, and 2- to 7-ppm S measured in AMB14-4; Ambenali Formation). Intraformational variability is consistent with previously observed variations of other geochemical characteristics (such as major elements or isotopes) among flows of the same formation (fig. S1B) (*6*, *34*, *35*, *37*; *42*–*45*), especially for lavas of the Kalsubai and Lonavala Subgroups. Nineteen of the 353 acquired S data are discarded as outliers, either on petrologic basis (they would be in equilibrium with a melt containing more sulfur than predicted by the sulfur concentration at sulfide saturation; SCSS) (*46*) or on statistical basis (they are local outliers in a population of homogeneous data; details in Supplementary Text) (Fig. 2C). The outliers are not considered when calculating and discussing S concentrations in the equilibrium melts (Fig. 4).

We measured fluorine in 34 clinopyroxenes from 21 basalts by secondary ion mass spectrometry (data S3) (*33*). The concentration of F in the crystals varies from 11 to 537 ppm (Fig. 3), but most data are in the range of 40 to 250 ppm (119 of 127 analyses). Some of the highest fluorine concentrations (up to 352 ppm) are found in clinopyroxenes from the Jawhar flows. Moving up through the sequence, fluorine concentrations drop in the upper Thakurvadi lavas (maximum of 85 ppm) and yield low values throughout the Lonavala Subgroup flows (maximum of 164 ppm, with the exception of one lower Khandala sample reaching 192 ppm), the Poladpur Formation (maximum of 75 ppm) and the base of Ambenali (maximum of 83 ppm). In the upper part of the section, from the top of Ambenali through the Mahabaleshwar Formation, clinopyroxenes have highly variable F concentrations (from 11 to 537 ppm). Whereas the two Jawhar samples show consistently high F, there is a large intraformational variability observed in lavas from the Ambenali (AMB14-4: 67- to 83-ppm F versus AMB14-8: 422- to 537-ppm F) and the Mahabaleshwar Formation (D231: 11 to 34 ppm versus PAS14-1: 63 to 222 ppm). Unlike sulfur, we do not observe intrasample variations for fluorine, most likely explained by the fact that this element is substantially more compatible with clinopyroxene than S (*32*, *33*) and is less prone to early degassing (*47*).

In summary, we observe that sulfur concentrations are relatively high in clinopyroxenes of the basal flows (Kalsubai Subgroup) and peak in crystals from the lavas of the Lonavala Subgroup, right before the KPB boundary (<0.10 Ma) as placed by ^40^Ar/^39^Ar geochronology, or ~0.10 Ma to 0.15 Ma before the KPB following the U-Pb age model (*3*, *24*, *41*). Then, sulfur concentrations become generally low in pyroxenes of the Wai Subgroup. Fluorine concentrations are highest in crystals from the base of the lava pile (Jawhar Formation) and near the top (high Ambenali Formation, but with large variability), whereas the remaining clinopyroxenes are generally F-poor, except for a few outliers. In general, S and F concentrations in clinopyroxenes are not correlated with each other across stratigraphy, if we exclude basalts of the Jawhar Formation, which are enriched in both volatiles (Fig. 4).

### Sulfur concentrations calculated for equilibrium melts

The in situ measurements allowed the calculation of the volatile concentrations in equilibrium melts based on recent determinations of sulfur partitioning between clinopyroxene and melt (data S3 and S7) (*31*, *32*). We used a clinopyroxene/melt S partition coefficient of 0.025 ± 0.002 (1 SD about the mean), suitable for basaltic rocks (fig. S1 and data S1). The maximum calculated S concentration in melts coexisting with clinopyroxene is 1769 ± 285 ppm (Fig. 4B), excluding the outliers (fig. S8A).

Sulfur is easily degassed from subaerially erupted basaltic magmas, with a degassing efficiency of 64 to 90% (*48*–*53*). A recent model of S degassing during magma ascent (*53*) demonstrates that H_2_O-poor basalts with low initial sulfate sulfur/total sulfur ratios (<0.1), similar to WG magmas, exsolve negligible amounts of sulfur except at very low pressures (<15 MPa). After this threshold, degassing is very efficient. Geobarometry suggests that the analyzed Deccan clinopyroxenes crystallized in the shallow continental crust (0.1 to 0.4 GPa) (fig. S3C), i.e., they offer a close portrait of the volatile budgets of the magmas upon eruption. Thus, maximum S concentrations calculated for the equilibrium melts can be considered as close approximations to the original sulfur concentrations of the magma during clinopyroxene crystallization, although not directly as degassed budgets (data S4). Conversely, very sulfur-poor crystals (<10 ppm) offer ambiguous information because their equilibrium magmas could be (a) originally poor in S, (b) sulfur-depleted due to previous removal of sulfides, or (c) degassed before clinopyroxene crystallization. Circumstance (a) is unlikely, as undegassed basaltic magmas, including flood basalts from LIPs, are usually enriched in dissolved sulfur (ca. 1500 ppm) (*31*, *51*–*53*). The negative correlation between MgO and Cu in Deccan Traps lavas (fig. S9) suggests that chalcophile elements behaved incompatibly, which is inconsistent with substantial sulfide removal [hypothesis (b)] (*40*). Plus, the presence of sulfides should buffer the melt at the SCSS, and any sulfur depletion should follow the evolution of the SCSS with changing melt composition (*54*). Case (c) is more likely; following the model of (*53*), this would mean that some clinopyroxenes crystallized in very shallow reservoirs (less than ~4-km depth), in agreement with the results from geobarometry (fig. S3C). Overall, the lack of correlation between S concentrations and major element crystal chemistry (figs. S4 to S6) and the mostly uniform S concentrations recorded along core-to-rim traverses (but see exceptions in fig. S4, E to G) argue against progressive degassing or S increase during fractional crystallization as the main explanation for intrasample variations. The ranges in sulfur recorded within the same sample may be due to crystallization from variably crustal contaminated melts at different depths or to the incorporation of phenocrysts from different reservoirs across a transcrustal plumbing system (*20*, *44*).

The maximum amount of sulfur that magma can dissolve before sulfides start to crystallize and sequester S from the melt provides an upper bound to the concentrations of sulfur calculated for equilibrium melts. The calculated SCSS (*46*) varies from 1100 to 2000 ppm in the investigated lavas (Fig. 4B). We calculated the SCSS for a clinopyroxene liquidus temperature of 1200°C and a pressure of 0.3 GPa, and oxygen fugacities near and slightly below the fayalite-quartz-magnetite buffer, as commonly observed for the Deccan Traps (*43*). The sulfur concentrations we calculate for the equilibrium melts are generally lower than, or similar to, the SCSS for the same melt compositions (Fig. 4B), in agreement with previous observations (*40*). The Bushe lava sample AMB14-1 is an exception, with 9 of 11 data points registering very high S concentrations, in equilibrium with melt S concentrations that exceed the SCSS. This sample shows some small sulfides in the mesostasis, but not in the silicates or in fractures (fig. S7C); therefore, it is unlikely that all the analyses targeted sulfides or secondary phases. More likely, this lava was supersaturated in sulfur (concentration in the melt exceeding 1000 ppm), due to either crustal contamination or a higher oxidation state (see Supplementary Text). Part of the sulfur in the melts from which the augites crystallized may have a crustal origin, especially since we found the highest S values in crystals coming from lava formations such as the Bushe, bearing the strongest evidence of crustal contamination (*6*, *7*, *34*, *35*, *40*), e.g., high whole-rock Th/Nb (fig. S10). Basaltic glass with S concentrations higher than SCSS has been measured in nature (*54*) and in experiments (*55*) in cases of crustal contamination with S-rich lithologies, such as organic matter-rich shales (up to ~10 wt % S) (*56*). However, isotopic and geologic evidence supports the assimilation of S-poor lithologies by the Bushe lavas (granitoids, tonalites, and amphibolites; S ca. 280 ppm) (*34*, *40*). In the absence of isotopic constraints on our targeted samples, we tentatively suggest the possibility that part of the S budget of this lava was derived from magma-crust interaction.

Overall, maximum S concentrations peak in the Jawhar Formation (KAS14-1, 1441 ± 232 ppm; the base of the lava pile) and in the Bushe Formation (sample AMB14-1, 1897 ± 306 ppm; sample KAM14-1, 1104 ± 178 ppm), preceding the KPB (Fig. 4B) (*1*–*3*, *24*, *41*). Maximum melt S concentrations calculated from clinopyroxenes of Jawhar (1441 ± 232 ppm) and Thakurvadi (780 ± 126 ppm) Formations overlap well with those previously measured from melt inclusions in plagioclase [ca. 1400 and 600 ppm, respectively (*28*)] (Fig. 4B). Sulfur concentrations calculated for Neral Formation samples (89 ± 34 to 389 ± 126 ppm) are for the most part lower than those previously reported for plagioclase- and olivine-hosted melt inclusions in this formation [400 to 1400 ppm (*28*)] (Fig. 4B). S concentrations measured in rare glass selvages preserved in one Neral Formation lava flow (*28*) range from 200 to 500 ppm, consistent with the equilibrium melts calculated from some clinopyroxenes, which are therefore possibly crystallized from partially degassed magmas.

We observe an evolution of the sulfur magmatic budget with stratigraphy, with sulfur-rich magmas (concentrations close to the SCSS) feeding Kalsubai Subgroup lava flows, and even more so for Lonavala Subgroup lavas (concentrations at or above the SCSS). Previously published melt inclusion data broadly support this picture. Our data suggest that the magmas that fed the Wai Subgroup eruptions were poor in S. This does not also imply that their parental magmas were S-poor. Sulfur degassing may be efficient if the lavas stall at very shallow depths [1 to 3 km, or even deeper (*52*)] for a relatively long time, and Wai lavas are generally evolved [MgO mostly less than 4 wt % (*6*, *34*, *35*)], a characteristic consistent with efficient fractionation processes and thus possibly also with efficient (passive) degassing.

### Fluorine concentrations calculated for equilibrium melts 

We calculated fluorine concentrations in equilibrium melts using individual partition coefficients calculated for each sample (*33*), based on representative compositions of the analyzed clinopyroxenes (D_F_^cpx/melt^ = 0.111 to 0.143; uncertainty ±25%, 1 SD about the mean for the regression line) (data S3 to S6). Fluorine concentrations in the equilibrium melts range from 80 ± 20 ppm to 3923 ± 983 ppm (Fig. 4C), peaking in lavas from the Jawhar Formation (KAS15-2; 1184 to 2611 ppm) and in those from the Ambenali Formation (AMB14-8; 3079 to 3923 ppm). The maximum (and the mean) fluorine concentrations for each formation zigzag through the section, with a minimum in the Lonavala and early Wai Subgroup flows, erupted respectively before and after the KPB (Fig. 4C).

Fluorine is far less volatile than sulfur in silicate melts, with 30 to 52% degassing efficiency from erupted lava for F compared to 64 to 90% for sulfur (*48*–*50*; *58*, *59*). Therefore, low fluorine concentrations are less likely explained by degassing, and they should reflect an original fluorine-poor magma. The low crystallinity (<15%) of most of our samples (fig. S2), their similar MgO concentrations (5 to 8 wt %) (fig. S3B and data S1), and the relatively restricted range of clinopyroxene compositions (data S2) argue against fractionation enrichment solely explaining the F-enriched compositions.

We compare our calculated F concentrations in equilibrium melts to data from other LIP lavas and from recent basaltic rocks (Fig. 4C) because no previous fluorine measurements are available for the Deccan Traps magmas. Our calculated equilibrium melts span a large range of F concentrations, with the lowest ones overlapping data from the Laki lava field (Iceland), the most recent historical analog example of flood basalt eruption (300- to 700-ppm F) (*49*, *60*), and those from mid-ocean ridge basalts (*61*). The most enriched of our calculated F concentrations overlap with those from the early and late emplacement phases of the Siberian Traps, known to be particularly halogen-rich, also compared to other LIPs (*20*), with oceanic island basalts (*61*), and with volatile-rich alkaline rocks from the Virunga volcanic province (62).

It is intriguing to link the evolving F concentrations across lava stratigraphy with varying amounts of source contributions, given that the continental lithospheric mantle has been recognized as an important source of F, particularly for LIP magmatism [see, e.g., (*63*–*65*)]. Fluorine and TiO_2_ concentrations in Deccan magmatic clinopyroxenes roughly correlate (fig. S11A), as previously observed for mantle clinopyroxenes (*63*). High-Ti lava samples tend to host clinopyroxenes with the highest fluorine concentrations (fig. S11B), possibly due to lithospheric contributions to the Kalsubai and part of Wai magma types [see, e.g., (*7*)]. However, we see no straightforward correlation between the here presented F budgets and previously published Nd and Hf isotope data for WG lavas (fig. S12) (*45*) arguing against a simple mantle-source control on the Ti and F contents of the relatively evolved Deccan basalts studied here (*6*, *7*, *38*, *42*).

## DISCUSSION

### Fluorine-rich basalts and their potential for regional fluorination of the ecosystems

Volcanogenic fluorine is unlikely to exert a strong force on the global environment because it is only moderately volatile and easily scavenged by rain from the volcanic plume (*58*). Instead, there is historical evidence for local impacts of fluorine degassing at the eruptive vents, as readily deposited from the volcanic haze, such as acid rain, crop failure, and livestock poisoning after the 1783–1784 Laki eruption (*60*). Pollution of water reservoirs was reported as a consequence of Etna eruptions (*66*). Exposure to highly fluorinated waters and soils or plants has consequences such as bone and teeth structure modification (fluorosis) and can cause oxidative and metabolic stress in mammals (*67*, *68*). The fluorine concentration in Laki basalts was comparable with the lowest among fluorine concentrations calculated in our Deccan dataset (Fig. 4C), yet it was responsible for stark local effects (*60*). Regional fluorination of the ecosystems likely occurred throughout the emplacement of the WG lavas and particularly during the activity of the Jawhar, Ambenali, and Mahabaleshwar lava formations, although, to date, any local or regional paleontological evidence is absent.

### Sulfur volcanic fluxes and global climatic implications

Paleothermometry based on multiple proxy data from marine and terrestrial records (Fig. 4A) reveals global warming in the Late Cretaceous (Late Maastrichtian) (3 to 4 K; 2 K on average) (*14*–*16*, *69*, *70*), beginning ~0.4 Ma before the KPB, coincident with volcanism in the northern Deccan and the onset of volcanism in the WG and MDVP. This warming phase peaked at ~0.2 Ma before the KPB and was followed by a steady global cooling event, ending at or just before the KPB, i.e., during the eruption of the Lonavala Subgroup (*1*, *2*) or immediately after it (*3*, *24*, *41*). The beginning of the Paleogene, contemporaneous with the emplacement of the voluminous Wai Subgroup lavas, marks a renewed trend of increasing temperatures (ca. 2 K), but at a lower rate than seen before the KPB (*14*–*16*, *69*, *70*).

Late Cretaceous global temperature changes might be induced by the Deccan Traps volcanogenic degassing, but the erupted volumes as reconstructed from the WG composite section do not seem to support this scenario (*36*). The Late Cretaceous warming-cooling doublet took place during the eruption of only the first 25 to 50% of the Deccan volume estimated from the WG lava pile, whereas half, if not the majority, of the erupted volume (50 to 75%) postdates the KPB and seems associated with a relatively minor temperature perturbation (*36*, *23*). Assuming that volumes reconstructed in the WG are representative of the entire province (*24*, *36*, *37*), the observed mismatch can be explained by different volatile budgets, eruptive styles, or emplacement rates between pre-KPB and post-KPB volcanism in the Deccan Traps (*14*).

Our dataset suggests a time-related change in volatile budgets through the emplacement of the WG lava pile, with magmas feeding eruptions of the pre-KPB Kalsubai and Lonavala Subgroups relatively rich in sulfur, halogens [as supported also by Self *et al*. (*28*)], and CO_2_ (*23*), and sulfur-poor magmas feeding post-KPB Wai Subgroup eruptions. Volatile-rich lavas were likely able to feed higher explosivity eruptions in particular at the onset of each volcanic pulse (*44*), with sustained energetic fountains or plumes capable of injecting SO_2_ into the stratosphere (*71*, *72*). Overall, the emplacement of volatile-rich pre-KPB formation lavas in the MDVP could have had a noticeable atmospheric impact, despite their lower volume compared to post-KPB Deccan volcanics.

Historical examples show that short-term cooling pulses can be provoked by sulfate aerosols formed by stratospheric SO_2_ discharge from single basaltic volcanic eruptions of medium to high explosivity, such as Laki (*60*). Recent models predict the sharpening of this effect in the presence of halogens (*21*). In the geologic past, short cooling episodes driven by SO_2_ discharge were modeled for the Deccan (*22*) and Siberian Traps LIPs (*73*). However, terrestrial stratigraphic records (*25*, *26*, *73*, *74*) lack the necessary time resolution to be capable of resolving such cold intervals because they are too brief. The marine records on the other hand are buffered compared with the atmosphere and may be less likely to show high-frequency variations from episodic S injections into the atmosphere (*14*–*16*, *18*, *27*, *69*, *70*, *74*).

The steady drop in temperature during the latest Cretaceous coincides with the emplacement of the Thakurvadi to Bushe lavas, the ones showing the highest S concentrations [maximum of ~1800-ppm S, for a total eruptive volume estimated between 86,000 and 466,000 km^3^ (*36*)]. Despite this, volcanically discharged SO_2_ might not have been the sole cause of the observed global cooling trend unfolding for 0.2 Ma during the latest Cretaceous. The effect of SO_2_ on the atmosphere cannot be linearly upscaled because of limiting factors to the conversion of SO_2_ to sulfate aerosols, such as water vapor in the atmosphere (*75*) or residence time in the stratosphere [1 to 3 years (*76*, *77*)]. However, as modeled before (*22*), sulfur emissions from Deccan Traps could have had marked consequences on the global climate (up to 10 K temperature drop) if discharged through sustained, prolonged activity (magmatic fluxes of 300 to 480 km^3^/year) even accounting for short hiatuses between individual eruptions.

Reconstruction of magmatic fluxes for LIP events comes with large uncertainties, and quantifying the duration of time elapsed during magmatic hiatuses is one of the most challenging frontiers of LIP research (*20*, *30*, *41*). Short-term eruptive fluxes are generally much higher than fluxes calculated as averaged along the entire peak emplacement phase of a LIP (10 to 100 times higher in the case of the Deccan Traps) (*20*). Independent data [paleomagnetism, geochronology, and terrestrial mercury records (*25*, *26*, *41*, *70*, *78*)] support the eruption of Deccan through pulsed activity at eruption rates of 10 to 100 km^3^/year, as typical of LIP magmatism. Such voluminous, recurring eruptive pulses could have caused repeated global cold spells (*73*), with temperature drops of up to about 10 K, interspersed with rapid recovery episodes. Sulfur budgets reconstructed by this study to SO_2_ (4 to 10 Tg/km^3^) for Kalsubai and Lonavala Subgroup lavas support previous models [for reference, a value of SO_2_ (5 Tg/km^3^) was estimated by Fendley *et al*. (*25*) and Self *et al*. (*28*)], whereby early eruptions in the MDVP were sufficient to produce repeated cold shocks. The final blow on the biota was likely given by the Chicxulub impact, but our dataset indicates that volcanic-driven climate disturbance was already underway before the KPB, possibly driving a press-pulse extinction model (*17*). Deccan Traps volcanism set the stage for a global biotic crisis, repeatedly deteriorating environmental conditions by forcing recurring short volcanic winters during the ~0.4 Ma before the Cretaceous-Paleogene boundary.

## MATERIALS AND METHODS

### Sulfur measurements

Sulfur in the clinopyroxenes was measured by SXRF at beamline I18 of the Diamond Light Source synchrotron, UK (*79*). Silicon and sulfur in the crystals were measured under a helium atmosphere, with a 3-keV beam focused to 6 μm by 6 μm by Kirkpatrick-Baez mirrors. The fluorescence spectra of the samples (data S7) were acquired with a Vortex silicon drift detector. We quantified sulfur concentrations from the spectra with PyMca software (*80*), using the silicon concentration of the minerals as an internal reference for quantification. The SXRF analytical technique for sulfur was tested by analysis of two in-house clinopyroxene crystals (*32*). The detection limit is calculated to be approximately 1 ppm for SXRF analyses by two different methods (*81*, *82*). An analytical uncertainty of 14% relative (1 SD; standard error on counting statistics) for samples with 6 ppm and greater is calculated by error propagation (*82*), based on 10% relative uncertainty (1 SD; standard error on counting statistics) in electron microprobe analyses of S concentration in standard glasses and 10% area uncertainty (1 SD; standard error on the measurement) during peak fitting of SXRF spectra. The uncertainty in the measured sulfur increases substantially at concentrations lower than 6 ppm, reaching 38% relative (1 SD) at 2 ppm. The uncertainty on the D_S values is 8% relative and is calculated as the standard deviation (1 SD) about the mean of all the measured D_S values for clinopyroxenes in low fO_2_ basaltic experiments (*31*, *32*).

### Fluorine measurements

Fluorine concentrations were analyzed in two sessions (data S6) by ion microprobe (CAMECA IMS 1280 at Nordsim Laboratory, Stockholm Natural History Museum, Sweden) using glass standards for calibration (*83*). The analytical protocol is based on (*84*) for halogen concentration measurements in phosphates and was modified by removing the Br and I measurements (*33*). A Gaussian-focused ^133^Cs^+^ primary beam of ~0.5 nA with an accelerating voltage of 10 kV was used. Low-energy normal-incidence electron flooding counteracted charge buildup on the target. At the beginning of each analysis, the primary beam was rastered over a square area of 20 × 20 μm for 120 s before data acquisition to remove the gold coating and surface contamination, and then reduced to a ~10-μm raster during data acquisition. Secondary ions were accelerated using a potential of 10 kV, centered in the field aperture, and optimized for mass calibration using the ^18^O signal. Secondary ion species at a mass resolution (*M*/Δ*M*) of 2430 were then measured by magnet peak switching on a low-noise, ion-counting electron multiplier. Ratios were made to ^18^O and working curves determined from regularly interspersed analysis of reference materials. The F working curve used the ATHO-G glass as the primary calibration reference and TI-G as a secondary monitor (F concentrations, 1006 and 233 ppm, respectively; E. Rose-Koga, Université Blaise Pascal, Clermont Ferrand, pers. comm.). A linear working curve was fitted through the origin. External precision (1 SD) based on measurements of ATHO-G was 1.1% in session 1 (*n* = 15) and 1.6% in session 2 (*n* = 28). This value was propagated together with the within-run uncertainty (observed SEM) to yield an estimate of overall uncertainty on the concentration measurements (excluding uncertainty in the reference material concentrations which is not well constrained in most cases). Treated as a secondary reference material, the TI-G glass yielded an F concentration of 220 ± 11 ppm (1 SD, *n* = 36 across both sessions), in good agreement with the expected value. A typical detector background between 0.005 and 0.01 counts per second yielded an F detection limit of 0.039 to 0.078 parts per billion. We note, however, that these are nominal detection limits based entirely on instrument parameters and do not take into account potential contributions from surface contamination and/or residual gasses in the sample chamber. Herasil glass is considered nominally F free although its F concentration has never been formally certified. Herasil glass was measured (*85*) on the CAMECA IMS 1280 at the Northeast National Ion Microprobe Facility (Woods Hole Oceanographic Institution) to constrain the effective maximum background value to 0.4-ppm F. We measured F on a different sample of Herasil glass. The average concentration we obtained for F was 2.1 ± 0.1 ppm (1 SD about the mean). Based on these measurements, we conservatively estimate 2.1-ppm F as our maximum background value for fluorine. The relative uncertainty (1 SD) for each single data point is reported in data S3. The D_F values were calculated starting from major element compositions and site occupancies of the targeted clinopyroxenes (data S5) (*33*). The uncertainty on this regression calculation is 25% relative (1 SD) about the mean. Error propagation on calculated equilibrium melt F concentrations yields 25% relative uncertainties (1 SD).

### Clinopyroxene and whole-rock major elements

Electron microprobe analyses on clinopyroxenes were performed at the CNR, IGG Padova (Italy), using a CAMECA SX50 electron microprobe. Natural and synthetic standards were used for instrumental calibration. Accelerating voltage and beam current were set respectively at 20 kV and 15 nA. ZAF online data reduction and matrix correction procedures were used. From frequent analyses of standards, relative analytical uncertainties are assessed at about 1% for major and 5% for minor elements (1 SD; standard error on counting statistics). Counting times were 10 s on the peak and background for Na, Al, and Si analysis, and 10 s on the peak and 5-s background for all other elements. Whole-rock major element and selected trace element contents were determined by x-ray fluorescence at University of Padova with a Philips PW2400 spectrometer, following previously described methods (*86*). Analytical uncertainties range from 1 to 2% (1 SD; standard error on counting statistics) for major elements and from 10 to 15% (1 SD; standard error on counting statistics) for trace elements.

## Supplementary Materials

### This PDF file includes:

Supplementary Text Figs. S1 to S12 Legends for data S1 to S7 References
