# Supplementary material for: Recurring volcanic winters during the latest Cretaceous: Sulfur and fluorine budgets of Deccan Traps lavas
Source: Sci Adv. 2023 Oct 4;9(40):eadg8284. doi: 10.1126/sciadv.adg8284 (PMC10550224; doi:10.1126/sciadv.adg8284)
Supplement: Supplementary file 1 — Supplementary Text Figs. S1 to S12 Legends for data S1 to S7 References [file sciadv.adg8284_sm.pdf]

Science Advances
AAAS

Supplementary Materials for

Recurring volcanic winters during the latest Cretaceous: Sulfur and fluorine budgets of Deccan Traps lavas

Sara Callegaro *et al.*

Corresponding author: Sara Callegaro, sara.callegaro@geo.uio.no

Sci. Adv. **9**, eadg8284 (2023)
DOI: 10.1126/sciadv.adg8284

The PDF file includes:

Supplementary Text
Figs. S1 to S12
Legends for data S1 to S7
References

Other Supplementary Material for this manuscript includes the following:

Data S1 to S7

Supplementary Text

Error propagation of analytical uncertainties on the equilibrium melt concentrations

The uncertainty on the calculated concentrations was then propagated as: $(\sigma Z/Z)^2 = (\sigma X/X)^2 + (\sigma Y/Y)^2$, where Z is the concentration of the element in the equilibrium melt, X is the concentration of the element in the clinopyroxene, and Y is the crystal/melt partition coefficient. Relative uncertainties on the calculated values for sulfur concentrations in equilibrium melts vary thus between 16 and 30% (1 SD) (fig. S8A). The relative uncertainty on the calculated fluorine concentrations in equilibrium melts is 25 % (1 SD) (fig. S8B).

Petrological and statistical outliers in the S-in-cpx dataset

Sulfur is contained in clinopyroxene as a trace element, and values of 100s-1000s ppm S are simply not conceivable to reflect S incorporation in cpx during crystallization at equilibrium from a melt [cf. values in ref. (31-32)]. Therefore, S>100 ppm is simply not equilibrium sulfur contained in the clinopyroxene structure (S-in-cpx), but in some other phase trapped by the crystal (microsulfides, fluid or melt inclusions), or in alteration phases in cracks, or in other miscellaneous contaminants upon the sample surface (synchrotron analyses are not carried out in vacuum but in a He-fluxed atmosphere). Only S-in-cpx values were suitable for calculation of S concentrations in equilibrium melts, hence any analysis was discarded upon showing clearly anomalous S peaks in the acquired spectra.

Of the collected spectra quantified by manual fitting with PyMCA, some still present suspiciously high S concentrations.

An anomalously high S data point can be discarded as petrological outlier, i.e. because translating into S concentration in the equilibrium melt above the SCSS for that lava (46, 54), or as a statistical outlier, i.e. the data point does not show anomalously high sulfur per se, but it is anomalous with respect to the sample (Fig. 2C) or the Formation (Fig. 4B) dataset. These anomalies highlight the importance of measuring S in multiple spots and in different crystals from the same lava sample, as well as in different samples from the same formation, to account for intra-sample and intra-formation variations, and avoid inferring province-wide concentrations from anomalously high or low data points [cf. ref. (31)].

Petrological outliers and the case of sample AMB14-1

One data point in cpx3 (fig. S2C) from sample D242 from the Mahabaleshwar Fm. (49±7 ppm S) (Data S3) [data from ref. (31)] translates into 1943±313 ppm S in the melt, while SCSS = 1305 ppm for this lava sample. Besides being a petrological outlier, this data point is also a statistical outlier.

One data point in cpx4 of sample MSJ14-8 (Khandala Fm.) shows a concentration of 148±21 ppm S, and is coupled with high counts for Cl (quantified automatically by PyMCA, no manual fitting) (Data S3 and S7). We interpret this data point as obtained from a fluid or melt inclusion. Melt inclusions in pyroxenes from this sample are visible by optical microscopy (cf. blue arrow in fig. S2A). This analysis was discarded because not relevant to the scope of this study (and as such, not included in the Figures). One point in cpx5 from the same sample shows 72±10 ppm S. This translates into 2885±465 ppm S in the melt, significantly above the calculated SCSS (1738 ppm). This lava sample does not present sulfides in the phenocrysts, nor in the mesostasis. Differently from the other anomalous data point found in cpx4 from this sample, no Cl enrichment

accompanies this spot analysis. This data point is discarded on petrological basis (red cross in Fig. 2C) and red font in Data S2. We tentatively explain it as resulting from hitting a crack or some impurity on the cpx phenocryst, which is indeed fractured (fig. S2B). Both these data points are also statistical outliers

One point in cpx2 from sample MAT14-6B (Khandala Fm.) (Data S3 and S7) shows 53±7 ppm S, translating to 2120±342 ppm in the melt, while SCSS = 1234 ppm. Therefore, we discard this data point on petrologic basis. This pyroxene is ophitic, enclosing plagioclase and an altered olivine. (fig. S2D). We hypothesize that the beam hit a crack, or one of the many enclosed crystals.

All the data points from Bushe sample AMB14-1(Data S2 and S7) show high S concentrations (from 25±4 to 72±10 ppm S), and the volcanostratigraphic position of this lava flow, immediately preceding the KPB, makes this worth of further discussion. All but 2 data points from AMB14-1 clinopyroxenes translate to S concentrations in the melt (from 999±161 ppm to 2861±461 ppm) that exceed the sulfur concentration at sulfide saturation (SCSS 999 ppm). The data points yielding ca. 70 ppm S are both petrologic and statistical outliers. The remaining 9 data points are here discussed.

AMB14-1 lava is slightly more SiO_2-rich than the others in the sample set (basaltic andesite, SiO_2 = 53 wt.%), consistent with the observation that lavas of the Bushe Fm. are generally the most contaminated by the crust in the Deccan LIP [e.g. (38)]. We tried to apply a higher clinopyroxene/melt D_S for this sample (0.032, suitable for andesitic compositions) (36), but even so most of the calculated S concentrations would still exceed the calculated SCSS (999 ppm).

Clinopyroxenes from Bushe sample AMB14-1 might be recording a transient condition of supersaturation in sulfur, possibly due to extra sulfur from an assimilated crustal melt, in which case (micro- or nano-) sulfides might have been crystallizing along with pyroxene. This is confirmed petrographically, since a few sulfides are visible in thin section. However, the sulfides observed in AMB14-1 are clearly interstitial, not included in augite crystals (fig. S7C). Basaltic glass with S concentrations higher than SCSS has been measured in nature (55) and in experiments (56) in case of crustal contamination with organic-rich shales, which are very rich in sulfur [up to ca. 10 wt.%; e.g. (57)]. However, isotopic evidence demonstrates that Bushe lavas assimilated S-poor lithologies (granitoids, tonalites, amphibolites, S ca. 280 ppm) (34, 40). Ref. (40) found that lavas of the Bushe formation are undersaturated in sulfides, despite their strongly contaminated nature. They specifically interpret this lack of sulfide saturation as prevented by the low S concentration of the assimilated lithologies.

Another possibility is that this particular Bushe magma was more oxidized than usual continental flood basalts, and capable of dissolving more sulfur in sulfate form (88-89), than that predicted as sulfide by the SCSS (46, 54), e.g., fO_2 1 log unit above the FMQ buffer. However, that would be unusual for Deccan volcanic rocks, which generally crystallized in T-fO_2 ranges broadly corresponding to the QFM buffer [e.g. (43)] and for LIP-related tholeiitic basalts in general (89). Unpublished data from oxides analyses of the collection of L. Melluso from a Mahabaleshwar flow and Tapti basalt dykes plot along the FMQ buffer on a T-vs.-fO_2 plot (L. Melluso collection). However, Bushe lavas are unusual, and warrant further research in this direction, because their contaminated nature could explain their more oxidized character.

Alternatively, the synchrotron beam hit a portion of the crystal that grew during anomalously rapid growth, which can lead to a disequilibrium increase in incompatible elements in the boundary layer immediately surrounding the crystal (known as “snow plow effect”; cf. studies on

apatite; 90-91). However, this is unlikely, since the targeted pyroxenes are small phenocrysts in an intersertal, glassy mesostasis, and they show textural and chemical equilibrium with the whole-rock (fig. S2E and S3B).

Similarly, if the augites were in disequilibrium with the melt, because they crystallized from a more primitive parental melt than the AMB14-1 whole-rock, i.e., before significant assimilation of crustal material, the calculated SCSS cannot be compared with the concentrations measured in the phenocrysts. However, textural and chemical observations (fig. S2E and S3B) which indicate equilibrium between these clinopyroxenes and the whole rock.

We favor the explanation that clinopyroxene crystals from Bushe sample AMB14-1 recorded a transient condition of supersaturation in sulfur, before or during the nucleation of sulfides, and in this sense the sulfur concentrations obtained in cpx by SXRF are relevant for this study (S-in-cpx), and translate into minimum S concentrations of 1000 ppm in this Bushe Formation magma.

<u>Sulfur concentrations measured along core-to-rim traverses (SXRF)</u>

We analyzed some of the crystals along traverses, with multiple spots (up to 48) in core-to-rim or rim-to-rim lines (Data S3, S7). The step-size was set between 6 and 11 μm. This was done to check for zoning in the S concentration and to investigate the existence of correlation between S and the major elements crystal chemistry (only FeO, MgO and CaO are plotted in Figs. S4-S6, the other clinopyroxene major element compositions are reported in Data S2).
To avoid inferring sample- or even formation-wide S concentrations based on anomalous points, the analysis of multiple points in a crystal, and of multiple crystals in a rock sample is recommended.

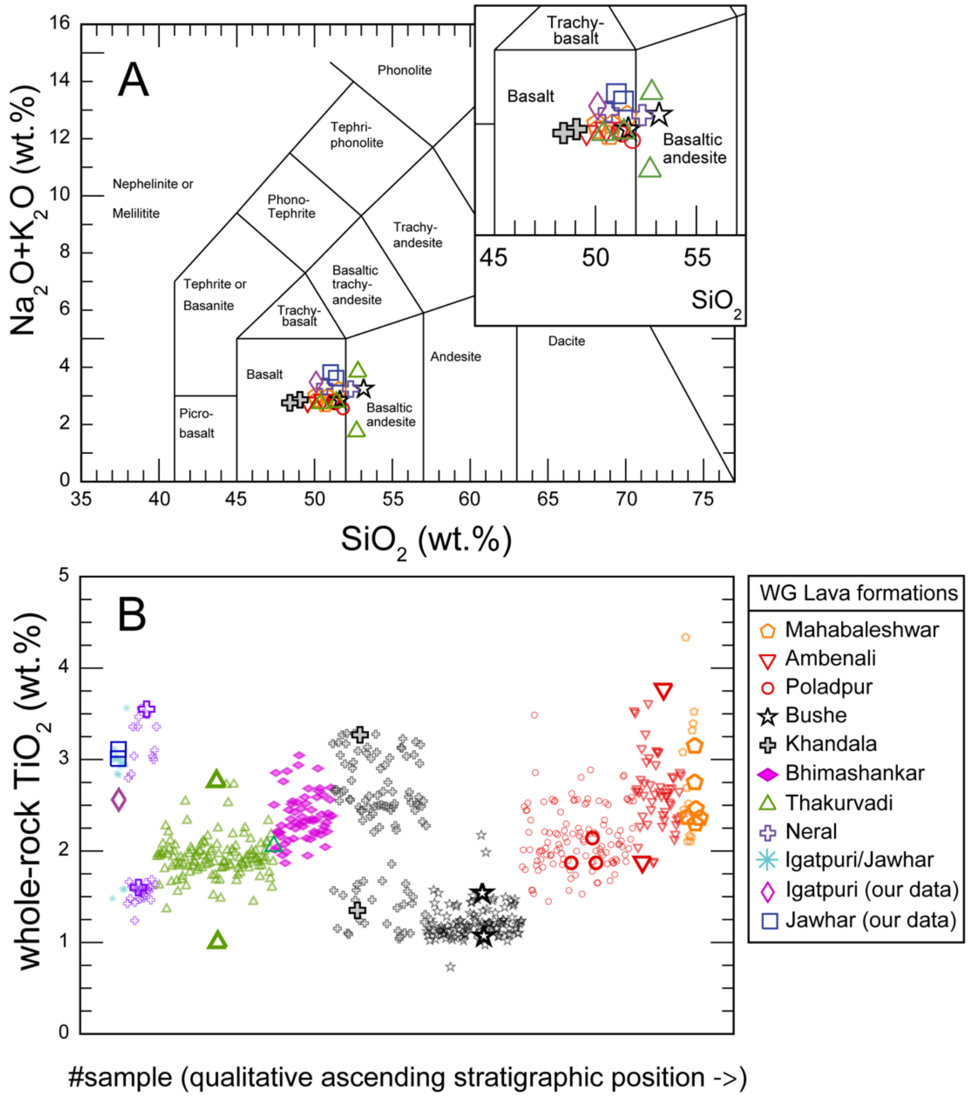

Fig. S1.

A) Whole-rock compositions of the investigated lava samples are plotted volatile-free on a TAS (Total Alkali Silica) (92) diagram. Enlarged inset included for clarity. Whole rock major and trace element data for the investigated Western Ghats lava samples are available in Data S1, including data from ref. (42) and data published here for the first time. Uncertainties are smaller than the data symbols. B) Whole-rock TiO_2 compositions of our data are plotted along with a partial compilation of published data from the Western Ghats lava pile (35). Our data are plotted as large symbols, compiled data as small symbols.

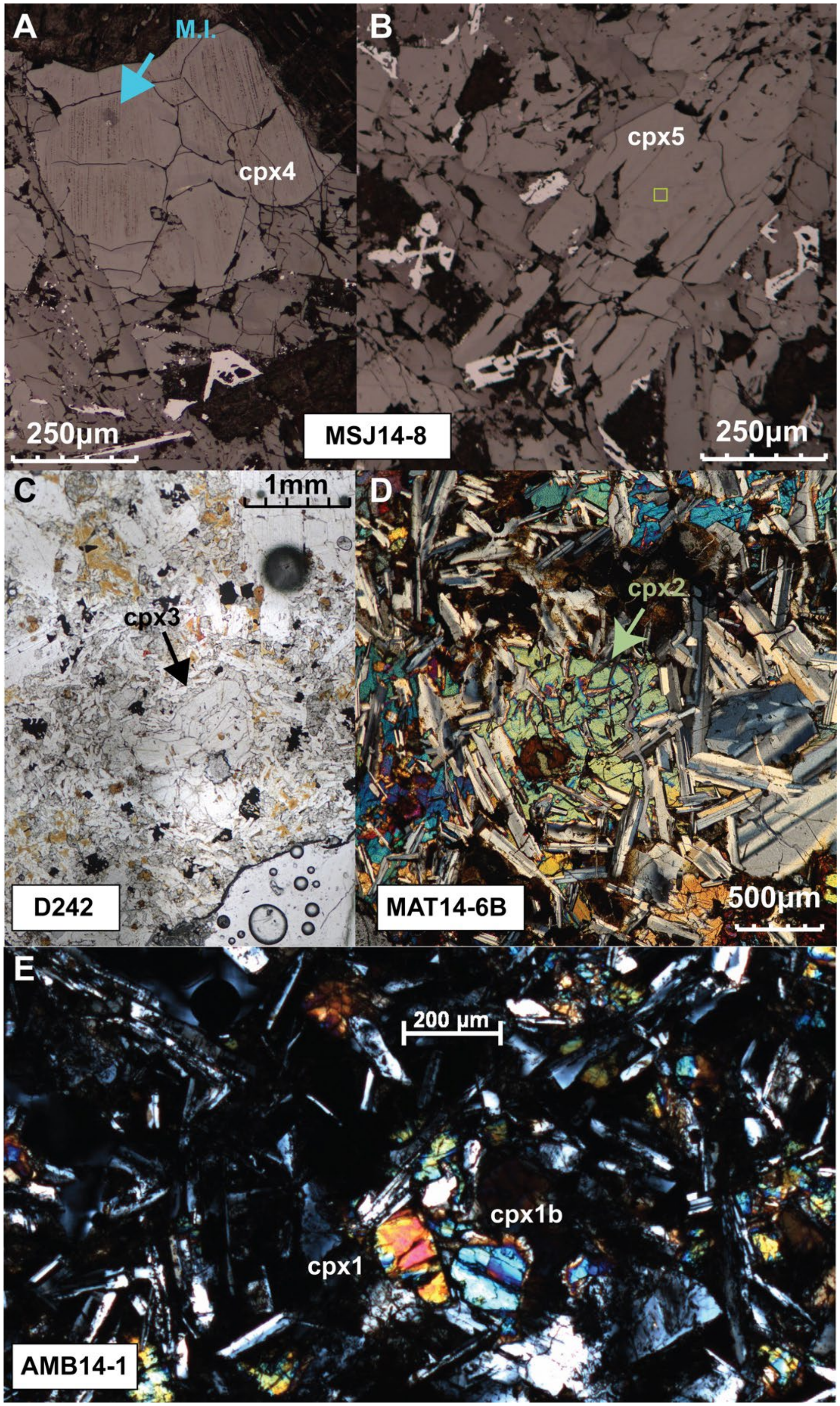

Fig. S2.

Photomicrographs of some the targeted clinopyroxenes showing anomalously high S concentrations by SXRF. Photos A and B are taken with reflected light, C with parallel polars and D and E with crossed polars. Note different scales. Blue arrow in A indicates a melt inclusion. Green square in B indicates the location of SXRF analyses in this clinopyroxene.

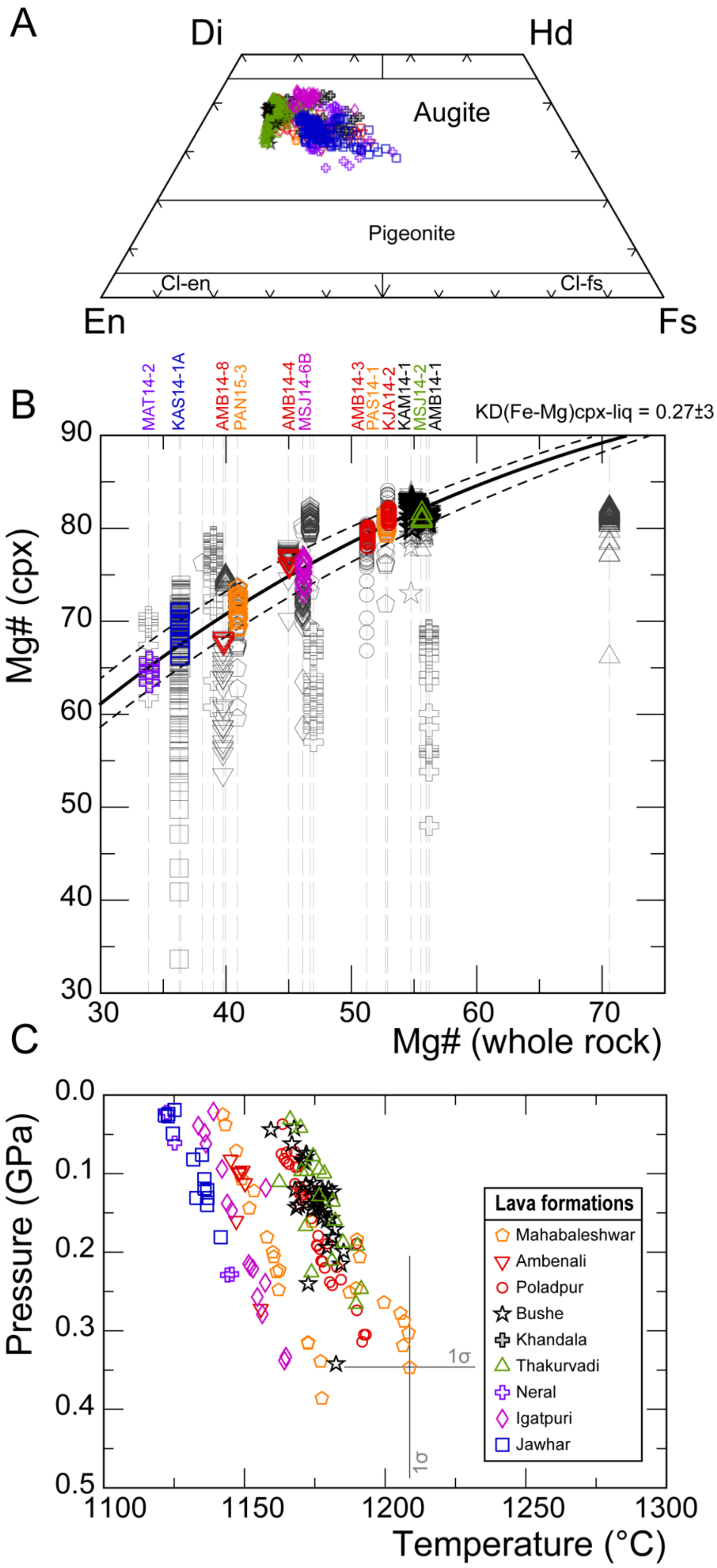
A
Di
Hd
Augite
Pigeonite
Cl-en
Cl-fs
En
Fs
B
MAT14-2
KAS14-1A
AMB14-8
PAN15-3
AMB14-4
MSJ14-6B
AMB14-3
PAS14-1
KJA14-2
KAM14-1
MSJ14-2
AMB14-1
KD(Fe-Mg)cpx-liq = 0.27±3
Mg# (cpx)
Mg# (whole rock)
C
Pressure (GPa)
Temperature (°C)
1σ
Lava formations
Mahabaleshwar
Ambenali
Poladpur
Bushe
Khandala
Thakurvadi
Neral
Igatpuri
Jawhar

Fig. S3. (previous page)

A) Clinopyroxene quadrilateral components of the targeted crystals (EMP analyses in Data S2). The major element compositions of the investigated clinopyroxenes fall within the augitic field as defined by the quadrilateral components Di (diopside, $CaMgSi_2O_6$), Hd (hedenbergite, $CaFeSi_2O_6$), En (enstatite, $Mg_2Si_2O_6$) and Fs (ferrosilite, $Fe_2Si_2O_6$), following the classification of ref. (93). Groundmass pigeonites and augites of the same samples were not plotted. The data points are color-coded per lava Formation (see legend in C). B) Clinopyroxene-whole rock equilibrium for the analyzed pyroxenes is shown on a Rhodes diagram. The equilibrium curves are built using a clinopyroxene/melt Fe-Mg K_D= 0.27±0.03 (94). Only clinopyroxene core compositions in equilibrium with host rocks were used to estimate crystallization pressures and temperatures. These are shown in colored symbols, the remaining compositions are reported in gray. Compositions above the equilibrium curve can represent xenocrystic pyroxenes; data plotting below the equilibrium curve (mostly rims of zoned clinopyroxenes) reflect late crystallized clinopyroxene and cumulus effect, as reflected also texturally by e.g. samples BOR14-2, MAT14-6B (fig. S2D), MAT14-1 (fig. S6). C) Crystallization temperature and pressure estimates for the investigated augites [(calculated following (95)]. The uncertainty is ±0.14 GPa (1 SD; standard error of estimate) in the calculated pressure, and ± 45 °C (1 SD; standard error of estimate) in the calculated temperature (95). For clarity, uncertainties are shown for one data point only.

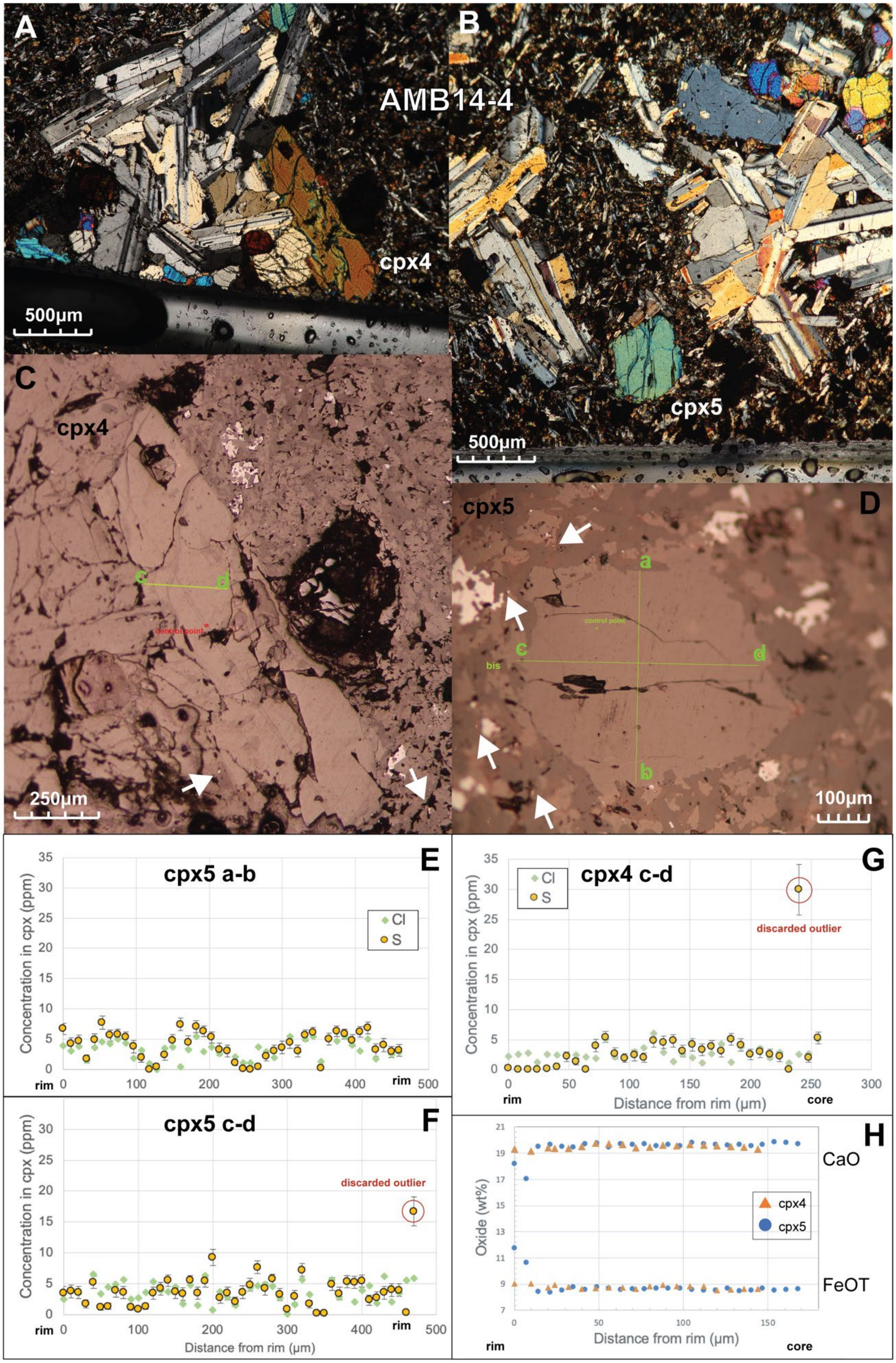

A
B
AMB14-4
cpx4
500µm
cpx5
500µm
C
cpx4
c
d
control point
250µm
D
cpx5
a
control point
c
d
bis
b
100µm
E
cpx5 a-b
Cl
S
Concentration in cpx (ppm)
rim
rim
G
cpx4 c-d
Cl
S
discarded outlier
Concentration in cpx (ppm)
Distance from rim (µm)
rim
core
F
cpx5 c-d
discarded outlier
Concentration in cpx (ppm)
Distance from rim (µm)
rim
rim
H
CaO
FeOT
cpx4
cpx5
Oxide (wt%)
Distance from rim (µm)
rim
core

Fig. S4. (previous page).

Photomicrographs of cpx4 and 5 of sample AMB14-4 (Ambenali Fm.) at crossed polars (A-B) and in reflected light (C-D). White arrows in C-D) indicate sulfides present in the lava sample, all are interstitial around the silicates, in the mesostasis. The green lines in C-D) mark the SXRF analytical traverses in cpx4 and 5, respectively. Sulfur and chlorine concentrations in cpx5 are plotted in E (line a-b) and F (line c-d). Sulfur and chlorine concentrations in cpx4 are plotted in G (line c-d). Chlorine concentrations are qualitative, based on automatic peak fitting. Uncertainties on the S concentration values are reported in Data S3.

Sulfur in cpx 5 varies from below detection limits to 10 ppm. Rhythmical zoning in S is visible in the rim-to-rim traverse a-b in cpx5 (E), but not in the orthogonal traverse c-d (F). S in cpx5 is not correlated with the major element variation (CaO and FeO_T wt.% as shown in H in blue circles). In H) CaO and FeO_T are uniform throughout cpx5, and show slight decrease and increase at the very rim, respectively. The EMP traverse follows ca. the a-b path reported in D). Concentrations of the other major elements for this crystal are reported in Data S2.

Low S concentrations (3-5 ppm) are visible for most of cpx 4 (G), and get below detection limit at the rim. S in cpx4 is not correlated with the major element variation (CaO and FeO_T wt.% as shown in H in orange triangles), which stay uniform throughout the crystal.

Two high S spots are found one at the rim and one at the core of cpx5 and 4, respectively, and we consider them as statistical outliers (that is, anomalous with respect to the rest of the dataset from the same crystal). Because they are not accompanied by concomitant high Cl concentrations, we interpret these local outliers as resulting from the beam hitting secondary microsulfides, for example in a crack. Major element concentrations (CaO and FeO_T; wt.%) are shown for the same crystals. The crystals show very uniform compositions except some perturbation at the very rim (FeO_T enrichment and CaO depletion). Note different scales.

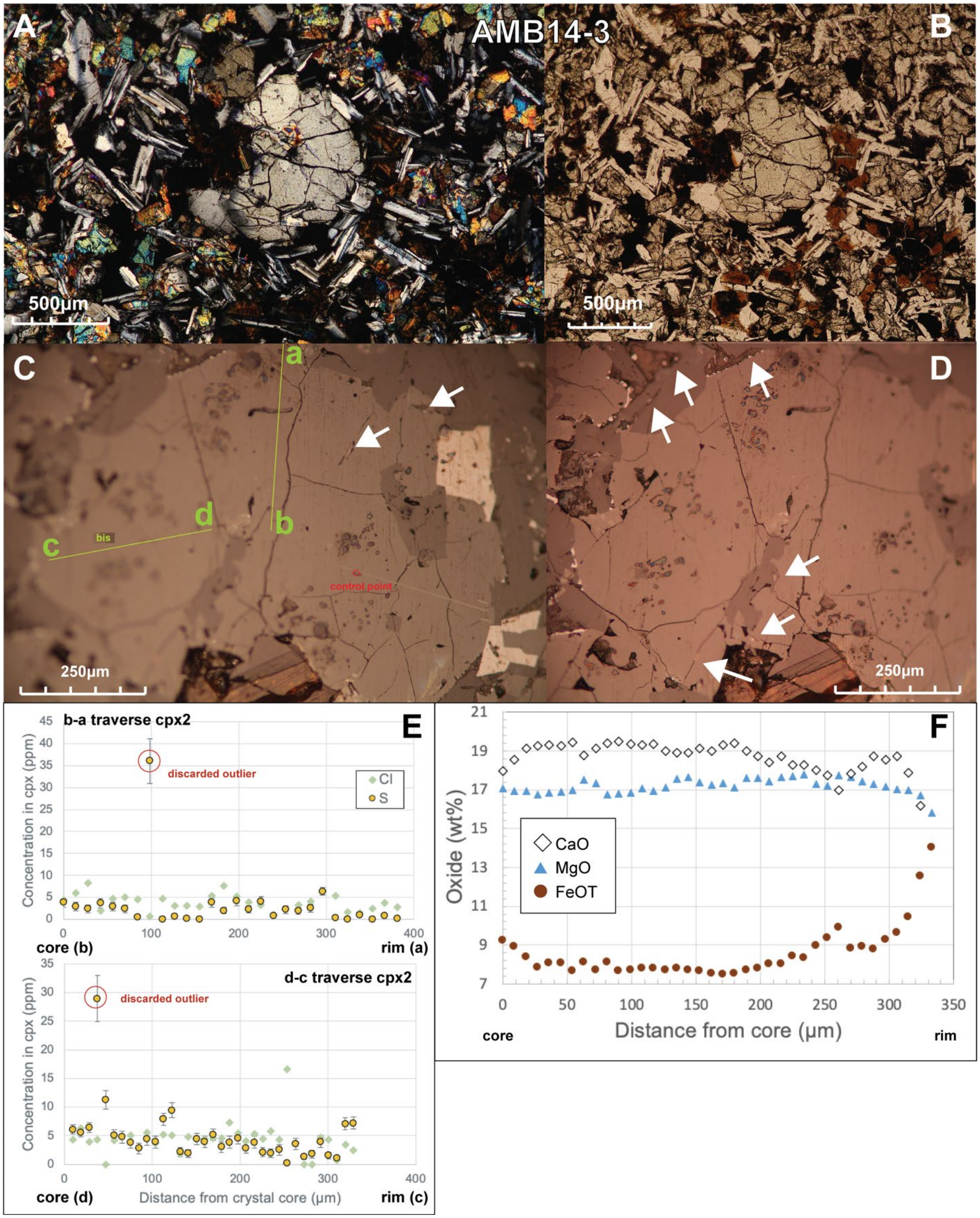

A
AMB14-3
B
500µm
500µm
C
a
b
d
c
bis
control point
D
250µm
250µm
E
b-a traverse cpx2
discarded outlier
Cl
S
Concentration in cpx (ppm)
core (b)
rim (a)
d-c traverse cpx2
discarded outlier
Concentration in cpx (ppm)
core (d)
Distance from crystal core (µm)
rim (c)
F
CaO
MgO
FeOT
Oxide (wt%)
Distance from core (µm)
core
rim

Fig. S5. (previous page).

Photomicrographs of cpx2 of sample AMB14-3 (Poladpur Fm.) at crossed polars (A), parallel polars (B) and in reflected light (C-D). White arrows in C-D) indicate sulfides present in the lava sample, all are interstitial around the silicates (in the mesostasis) or in cracks. The green lines in C) mark the SXRF analytical traverses in cpx2. Sulfur and chlorine concentrations in cpx2 are plotted in E (core to rim lines b-a and c-d). Chlorine concentrations are qualitative, based on automatic peak fitting. Uncertainties on the S concentration values are reported in Data S3. The major element (electron microprobe analyses) traverse follows b-a.

Two high S spots are found at the mantle and core of cpx2, and we consider them as statistical outliers (that is, anomalous with respect to the rest of the dataset from the same crystal). Because they are not accompanied by concomitant high Cl concentrations, we interpret these local outliers as resulting from the beam hitting secondary microsulfides, for example in a crack, as supported by textural evidence, or miscellaneous contaminants on the surface. The rest of the data population is fairly uniform (4±2 ppm). S in cpx2 is not correlated with the major element variation (CaO, MgO and FeO_{tot} wt.%) as shown in F). Major oxide concentrations (CaO and FeO_{tot}; wt.%) are shown for the same crystal. Slight normal zoning is visible, with increase in FeO_{tot} and decrease in CaO and MgO towards the rim. Concentrations of the other major oxides for this crystal are reported in Data S2. Note different scales.

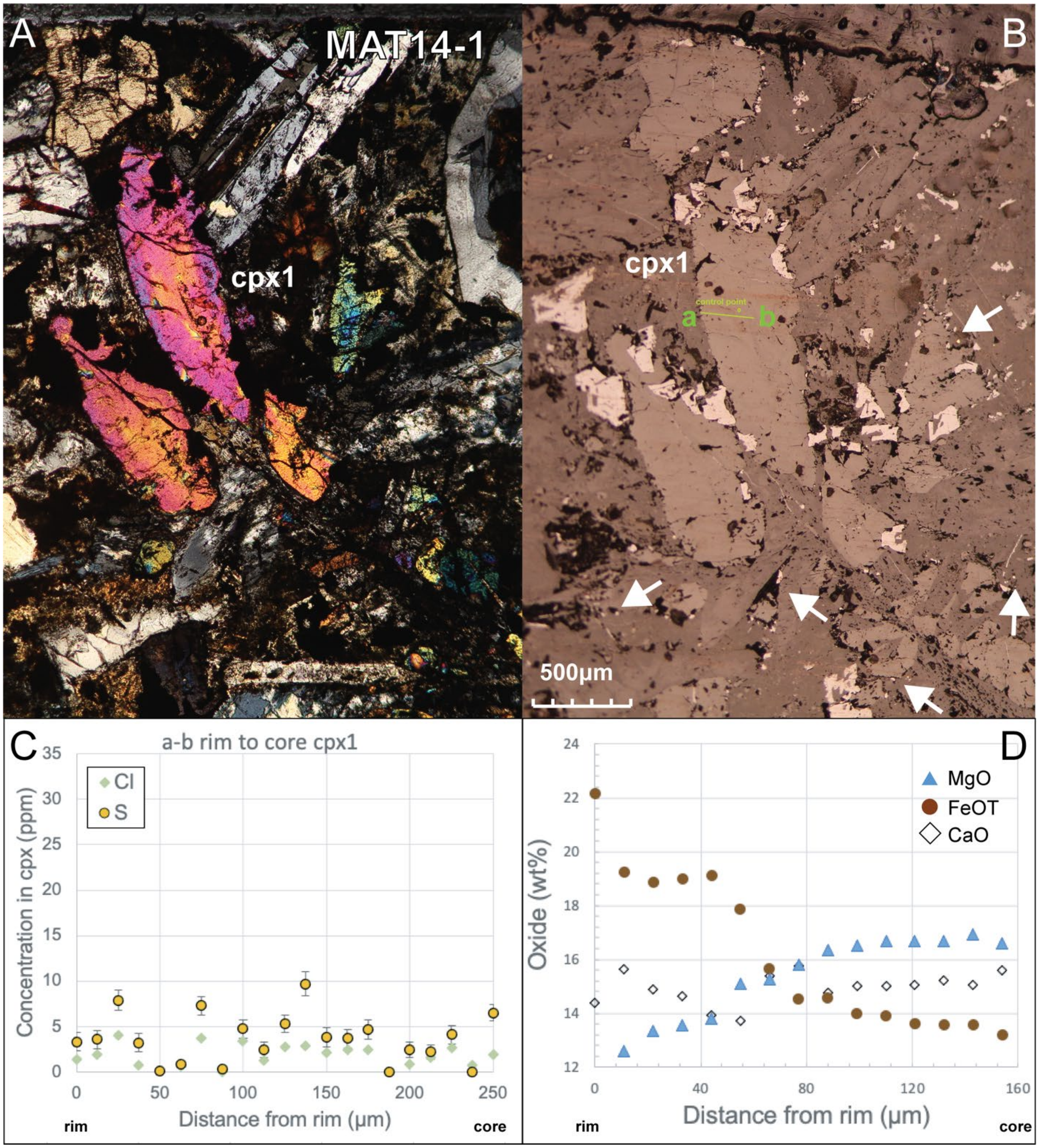
A
MAT14-1
cpx1
B
cpx1
a
b
500µm
C
a-b rim to core cpx1
Cl
S
Concentration in cpx (ppm)
Distance from rim (µm)
rim
core
D
MgO
FeOT
CaO
Oxide (wt%)
Distance from rim (µm)
rim
core

Fig. S6. (previous page).

Photomicrographs of cpx1 of sample MAT14-1 (Neral Fm.) at crossed polars (A) and in reflected light (B). White arrows in B) indicate sulfides present in the lava sample, all are interstitial around the silicates, in the mesostasis. The green line in B) marks the SXRF analytical traverse in cpx1, from which sulfur and chlorine concentrations in cpx are plotted in C). Chlorine concentration is only qualitative, based on automatic peak fitting. Uncertainties on the S concentration values are reported in Data S3. Sulfur in cpx 1 varies from below detection limits to 10 ppm, and is not correlated with the major element variation (normal zoning, increase in FeOtot and decrease in MgO) as shown in D). The EMP traverse follows ca. the a-b path reported in B) and major element concentrations (CaO, MgO and FeO_{tot}; wt.%) along this traverse are shown. Concentrations of the other major elements for this crystal are reported in Data S2. Note different scales.

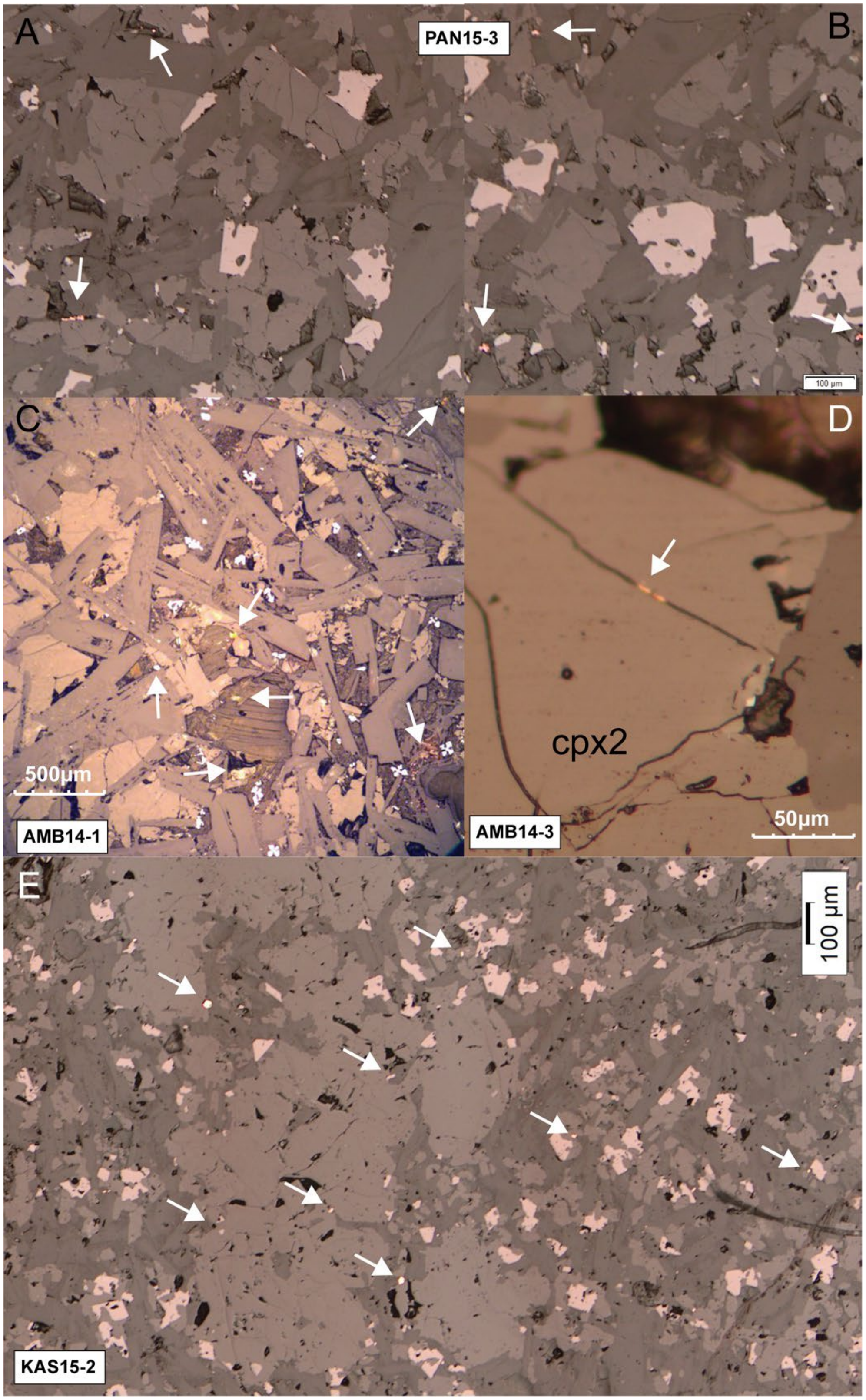

Fig. S7.
A-E) Photomicrographs of the targeted samples in reflected light. Sulfides are only found in a minority of the samples (7 out of 23 lavas) and only in very small amounts and size in the mesostasis or in cracks, i.e., interstitial with respect to the silicate phases. These sulfides are thus interpreted as secondary, or crystallized later than clinopyroxene. White arrows indicate the sulfides. Note different scales.

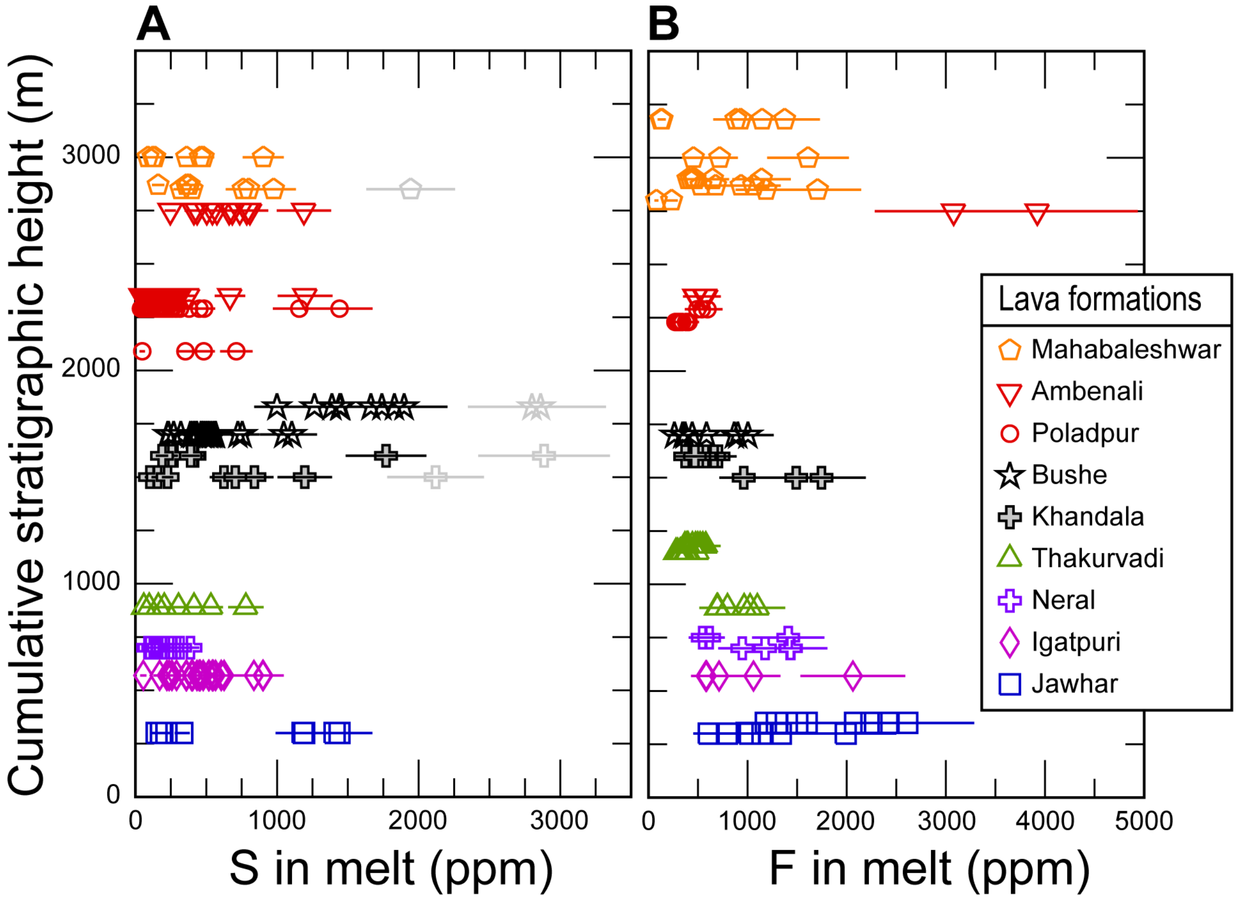

Fig. S8.
Concentrations of S (A) and F (B) calculated in melts in equilibrium with the analyzed clinopyroxenes are plotted in order of stratigraphic height and color-coded per Formation. Uncertainties on the calculated values are plotted as ±1 SD error bars and are between 22-31 % relative for S, and 25 % relative for F. In A) petrological outliers (n=5) are reported in gray (see Supplementary Text).

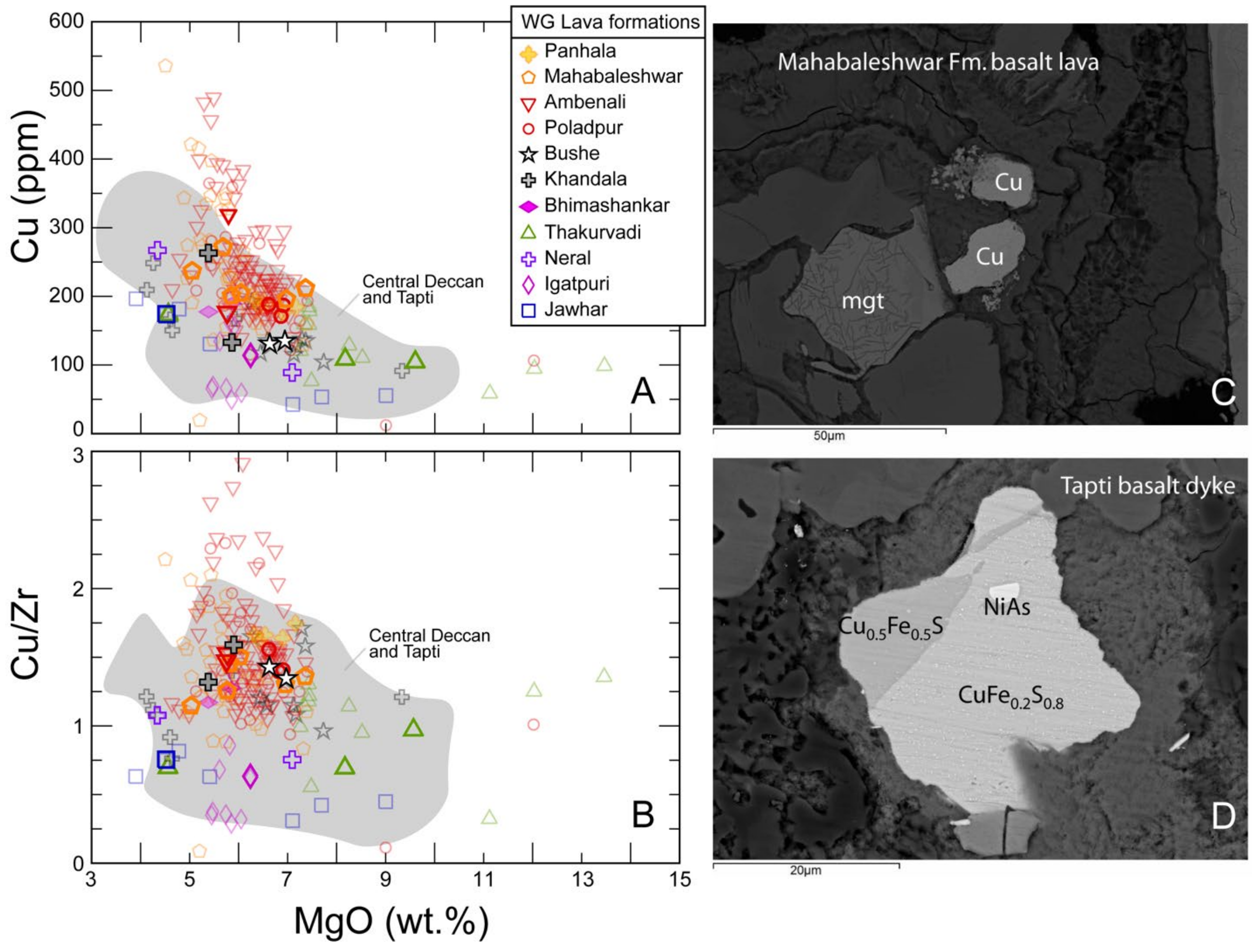

Fig. S9.

A-B) Plots of Cu (ppm) and Cu/Zr vs. MgO (wt.%) for the investigated samples (large symbols) and other Western Ghats lavas [data from refs. (40, 96-98)]. A compositional field for Central Deccan and Tapti volcanics is shown for comparison [data from refs. (98-99)]. The incompatible behavior shown by Cu and other chalcophile elements (40) argues against sulfide saturation of these lavas during their evolution. The strong enrichment in Cu in Wai group lavas is due to the presence of native Cu, as shown by the BSE image in C). Small sulfides found in the lavas (figs. S4-S7) are interstitial and most likely secondary, potentially hydrothermal (99), as suggested in D) by the presence of niccolite and chalcopyrite in a Tapti basalt dyke, shown here for comparison (BSE image from a sample of L. Melluso's collection).

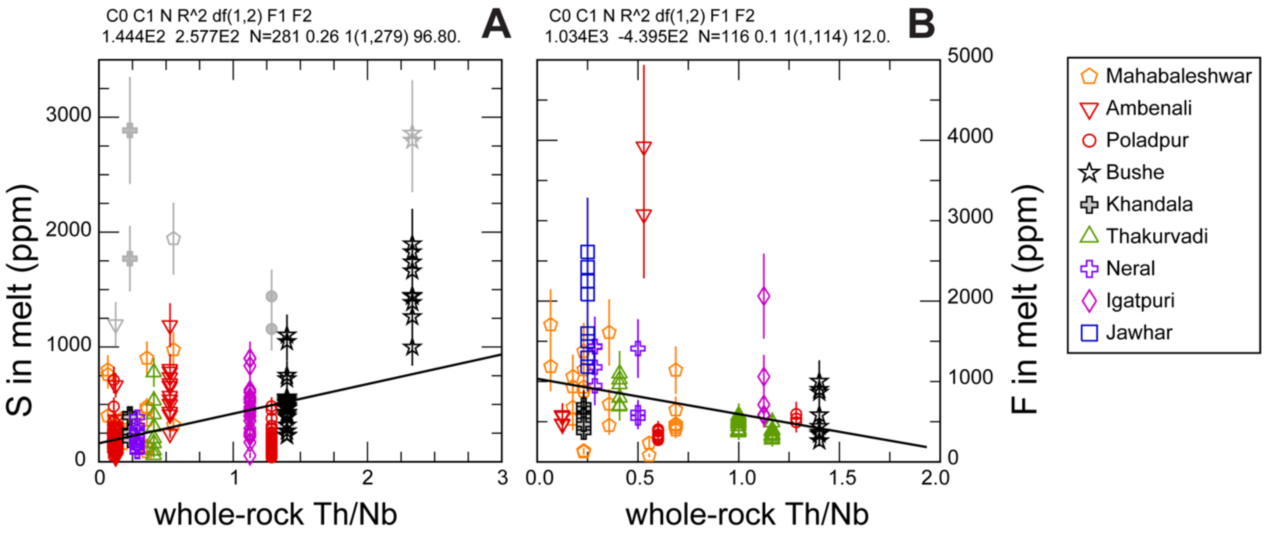

Fig. S10. S and F in the equilibrium melts are plotted along whole-rock Th/Nb as proxy for crustal contamination. (Data S1 and S3).

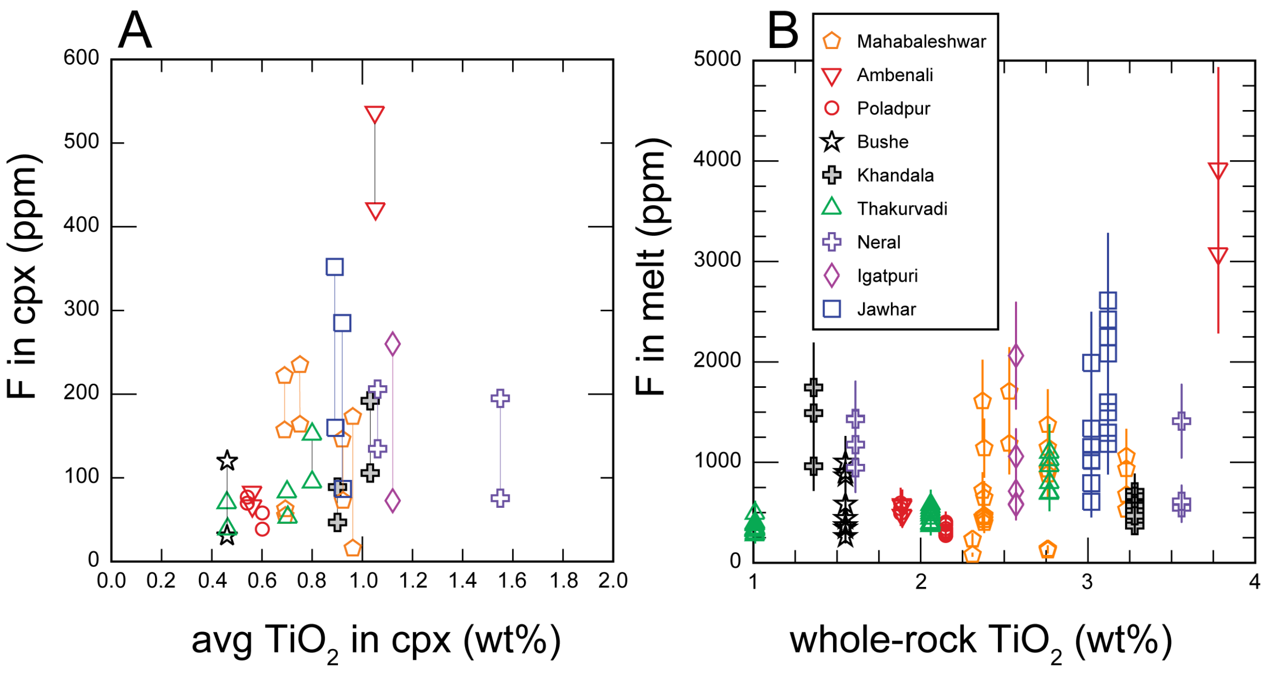

Fig. S11.

(A) Maximum and minimum fluorine concentrations (ppm) measured in clinopyroxene are plotted against the average concentration of TiO_2 (wt.%) in the clinopyroxene. (B) Fluorine concentrations (ppm) calculated in the equilibrium melt are plotted against the the whole-rock concentration in TiO_2 (wt.%). Data S1-S3.

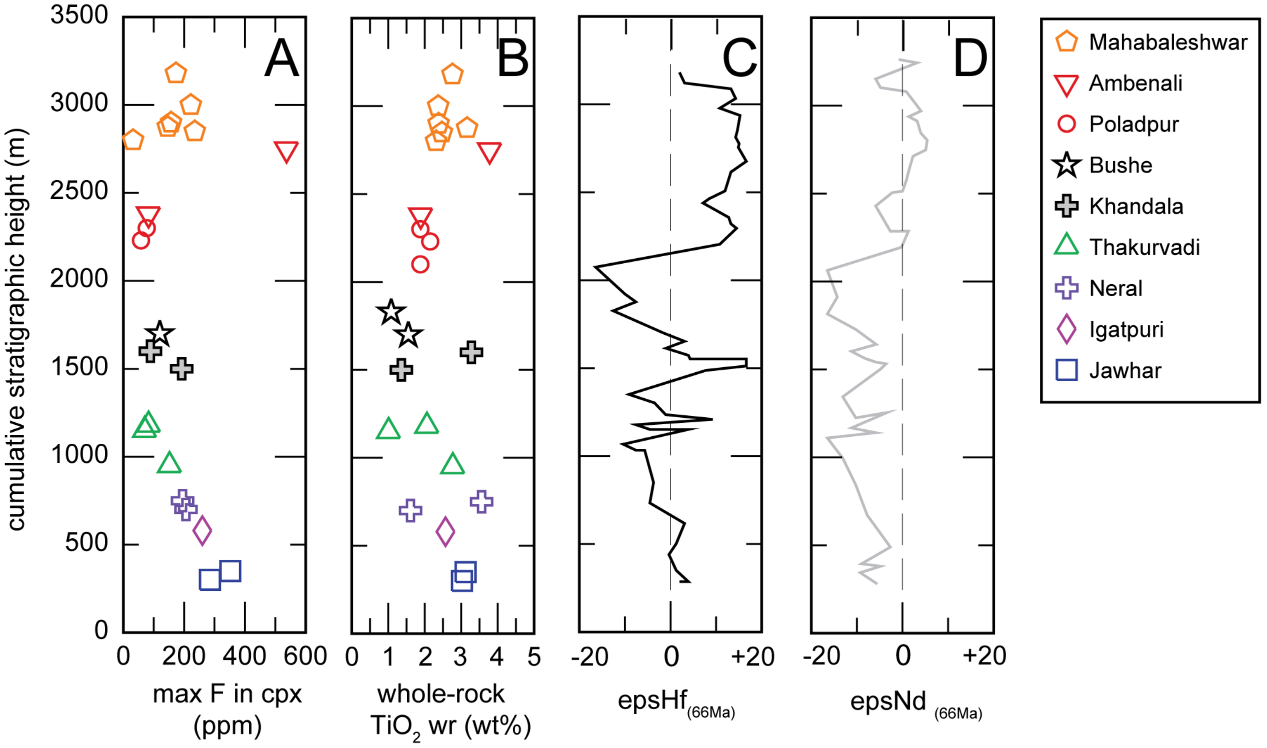

Fig. S12. Max F measured in clinopyroxene in ppm (A), whole-rock TiO_2 concentration in wt.% (B), and the whole-rock isotopic compositions reported as time-corrected εHf (C), and time-corrected εNd (D) are plotted in stratigraphic order (cumulative stratigraphic height) for the here studied samples (A-B) and for rock samples from ref. (45). The εHf and εNd values of the samples for the literature are connected by a continuous line. The position of the samples along the y-axis in C-D is adjusted from ref. (45) to match the Formation of pertinence in A-B to allow for comparison.

Data S1. (separate file)
Overview of the sample set. Whole-rock (wt%) and trace element (ppm) compositions are reported for the targeted lava samples along with a brief petrographic description of the textures and the presence or absence of sulfides. Ages (in Ma) are from ref. (1-2).

Data S2. (separate file).
Major element compositions (in wt%) of all the analyzed clinopyroxenes and other clinopyroxene phenocrysts in the same lavas. Pressure and Temperature estimates (95) are reported only for clinopyroxene compositions in equilibrium with the whole-rocks.

Data S3. (separate file).
Sulfur (synchrotron-light XRF) and fluorine (SIMS) concentrations in clinopyroxenes are reported in ppm. Each measured data point is accompanied by the relative analytical uncertainty. Calculated concentrations in the equilibrium melt (in ppm) are reported for each data point, except for the petrological outliers, are accompanied by absolute uncertainties (in ppm) obtained by combining analytical and partition coefficient uncertainties. The petrological outliers discussed in the Supplementary Text are reported in red. For each lava sample we report the cumulative stratigraphic height in the Western Ghats lava pile, the calculated clinopyroxene-melt partition coefficient for fluorine (33), the calculated sulfur concentration at sulfide saturation (SCSS) (46), and, when available, the high-precision plagioclase $^{40}Ar/^{39}Ar$ age in Ma (1-2).

Data S4. (separate file). Total sulfur and fluorine magmatic and degassed budgets are calculated for the Western Ghats Formations based on our dataset following the approach of (48). The eruptive volumes are taken from (36). Degassing rates are estimated based on these calculations coupled with high-precision $^{40}Ar/^{39}Ar$ ages (1-2).

Data S5. (separate file). Major element compositions (wt%) for the clinopyroxenes analyzed by SIMS are used to calculate the appropriate clinopyroxene/melt fluorine partition coefficient based on site occupancies (33).

Data S6. (separate file). Raw data from SIMS analyses, including standards.

Data S7. (separate file). Raw data from SXRF analyses.

REFERENCES AND NOTES

1. C. J. Sprain, P. R. Renne, L. Vanderkluysen, K. Pande, S. Self, T. Mittal, The eruptive tempo of Deccan volcanism in relation to the Cretaceous-Paleogene boundary. *Science* **363**, 866–870 (2019).

2. P. R. Renne, C. J. Sprain, M. A. Richards, S. Self, L. Vanderkluysen, K. Pande, State shift in Deccan volcanism at the Cretaceous-Paleogene boundary possibly induced by impact. *Science* **350**, 76–78 (2015).

3. B. Schoene, M. P. Eddy, K. M. Samperton, C. B. Keller, G. Keller, T. Adatte, S. F. R. Khadri, U-Pb constraints on pulsed eruption of the Deccan Traps across the end-Cretaceous mass extinction. *Science* **363**, 862–866 (2019).

4. J. J. Sepkoski, Patterns of phanerozoic extinction: A perspective from global data bases, in *Global Events and Event Stratigraphy in the Phanerozoic*, O. H. Walliser, Ed. (Springer, 1996).

5. M. J. Henehan, A. Ridgwell, E. Thomas, S. Zhang, L. Alegret, D. N. Schmidt, J. W. B. Rae, J. D. Witts, N. H. Landman, S. E. Greene, B. T. Huber, J. R. Super, N. J. Planavsky, P. M. Hull, Rapid ocean acidification and protracted Earth system recovery followed the end-Cretaceous Chicxulub impact. *Proc. Natl. Acad. Sci. U.S.A.* **116**, 22500–22504 (2019).

6. C. Beane, P. R. Turner, K. Hooper, J. Subbarao, J. N. Walsh, Stratigraphy, composition and form of the Deccan basalts, Western Ghats, India. *Bull. Volcanol.* **48**, 61–83 (1986).

7. P. Lightfoot, C. Hawkesworth, Origin of Deccan Trap lavas: Evidence from combined trace element and Sr-, Nd- and Pb-isotope studies. *Earth Planet. Sci. Lett.* **91**, 89–104 (1988).

8. L. W. Alvarez, W. Alvarez, F. Asaro, H. V. Michel, Extraterrestrial cause for the Cretaceous-Tertiary extinction. *Science* **208**, 1095–1108 (1980).

9. S. P. S. Gulick, T. J. Bralower, J. Ormö, B. Hall, K. Grice, B. Schaefer, S. Lyons, K. H. Freeman, J. V. Morgan, N. Artemieva, P. Kaskes, S. J. De Graaff, M. T. Whalen, G. S. Collins, S. M. Tikoo, C. Verhagen, G. L. Christeson, P. Claeys, M. J. L. Coolen, S. Goderis, K. Goto, R. A. F. Grieve, N. McCall, G. R. Osinski, A. S. P. Rae, U. Riller, J. Smit, V. Vajda, A. Wittmann, The first day of the

Cenozoic. *Proc. Natl. Acad. Sci. U.S.A.* **116**, 19342–19351 (2019).

10. G. Keller, Cretaceous climate, volcanism, impacts, and biotic effects. *Cret. Res.* **29**, 754–771 (2008).

11. P. Schulte, L. Alegret, I. Arenillas, J. A. Arz, P. J. Barton, P. R. Bown, T. J. Bralower, G. L. Christeson, P. Claeys, C. S. Cockell, G. S. Collins, A. Deutsch, T. J. Goldin, K. Goto, J. M. Grajales-Nishimura, R. A. F. Grieve, S. P. S. Gulick, K. R. Johnson, W. Kiessling, C. Koeberl, D. A. Kring, K. G. MacLeod, T. Matsui, J. Melosh, A. Montanari, J. V. Morgan, C. R. Neal, D. J. Nichols, R. D. Norris, E. Pierazzo, G. Ravizza, M. Rebolledo-Vieyra, W. U. Reimold, E. Robin, T. Salge, R. P. Speijer, A. R. Sweet, J. Urrutia-Fucugauchi, V. Vajda, M. T. Whalen, P. S. Willumsen, The Chicxulub asteroid impact and mass extinction at the Cretaceous-Paleogene boundary. *Science* **327**, 1214–1218 (2010).

12. S. V. Petersen, A. Dutton, K. C. Lohmann, End-Cretaceous extinction in Antarctica linked to both Deccan volcanism and meteorite impact via climate change. *Nat. Commun.* **7**, 1–9 (2016).

13. A. A. Chiarenza, A. Farnsworth, P. D. Mannion, D. J. Lunt, P. J. Valdes, J. V. Morgan, P. A. Allison, Asteroid impact, not volcanism, caused the end-Cretaceous dinosaur extinction. *Proc. Natl. Acad. Sci. U.S.A.* **117**, 17084–17093 (2020).

14. P. M. Hull, A. Bornemann, D. E. Penman, M. J. Henehan, R. E. Summons, E. Thomas, T. Westerhold, J. H. Whiteside, On impact and volcanism across the Cretaceous–Paleogene boundary. *Science* **367**, 266–272 (2020).

15. J. S. K. Barnet, K. Littler, D. Kroon, M. J. Leng, T. Westerhold, U. Röhl, J. C. Zachos, A new high-resolution chronology for the late Maastrichtian warming event: Establishing robust temporal links with the onset of Deccan volcanism. *Geology* **46**, 147–150 (2018).

16. V. Gilabert, S. J. Batenburg, I. Arenillas, J. A. Arz, Contribution of orbital forcing and Deccan volcanism to global climatic and biotic changes across the Cretaceous-Paleogene boundary at Zumaia, Spain. *Geology* **50**, 21–25 (2022).

17. N. C. Arens, A. Thompson, A. H. Jahren, A preliminary test of the press-pulse extinction hypothesis: Palynological indicators of vegetation change preceding the Cretaceous-Paleogene boundary, McCone County, Montana USA. *Spec. Pap. Geol. Soc. Am.* **503**, 209–227 (2014).

18. G. Keller, P. Mateo, J. Monkenbusch, N. Thibault, J. Punekar, J. E. Spangenberg, S. Abramovich, S. Ashckenazi-Polivoda, B. Schoene, M. P. Eddy, K. M. Samperton, S. F. R. Khadri, T. Adatte, Mercury linked to Deccan Traps volcanism, climate change and the end-Cretaceous mass extinction. *Glob. Planet. Change* **194**, 103312 (2020).

19. T. Green, P. R. Renne, C. B. Keller, Continental flood basalts drive Phanerozoic extinctions. *Proc. Natl. Acad. Sci. U.S.A.* **119**, e2120441119 (2022).

20. B. A. Black, L. Karlstrom, T. A. Mather, The life cycle of large igneous provinces. *Nat. Rev. Earth Environ.* **2**, 840–857 (2021).

21. J. Staunton-Sykes, T. J. Aubry, Y. M. Shin, J. Weber, L. R. Marshall, N. Luke Abraham, A. Archibald, A. Schmidt, Co-emission of volcanic sulfur and halogens amplifies volcanic effective radiative forcing. *Atmos. Chem. Phys.* **21**, 9009–9029 (2021).

22. A. Schmidt, R. A. Skeffington, T. Thordarson, S. Self, P. M. Forster, A. Rap, A. Ridgwell, D. Fowler, M. Wilson, G. W. Mann, P. B. Wignall, K. S. Carslaw, Selective environmental stress from sulphur emitted by continental flood basalt eruptions. *Nat. Geosci.* **9**, 77–82 (2016).

23. A. Hernandez Nava, B. A. Black, S. A. Gibson, R. J. Bodnar, P. R. Renne, L. Vanderkluysen, Reconciling early deccan traps CO_2 outgassing and pre-KPB global climate. *Proc. Natl. Acad. Sci. U.S.A.* **118**, e2007797118 (2021).

24. M. P. Eddy, B. Schoene, K. M. Samperton, G. Keller, T. Adatte, S. F. R. Khadri, U-Pb zircon age constraints on the earliest eruptions of the Deccan Large Igneous Province, Malwa Plateau, India. *Earth Planet. Sci. Lett.* **540**, 116249 (2020).

25. I. M. Fendley, T. Mittal, C. J. Sprain, M. Marvin-DiPasquale, T. S. Tobin, P. R. Renne, Constraints on the volume and rate of Deccan Traps flood basalt eruptions using a combination of high-resolution terrestrial mercury records and geochemical box models. *Earth Planet. Sci. Lett.* **524**, 115721 (2019).

26. S. Li, S. E. Grasby, X. Zhao, J. Chen, D. Zheng, H. Wang, Y. Fang, Q. Zhang, T. Yu, J. Tian, S. Du, E. A. Jarzembowski, Q. Wang, H. Zhang, X. Wan, B. Wang, Mercury evidence of Deccan volcanism driving the Latest Maastrichtian warming event. *Geology* **50**, 1140–1144 (2022).

27. E. Font, T. Adatte, A. N. Sial, L. D. de Lacerda, G. Keller, J. Punekar, Mercury anomaly, Deccan volcanism, and the end-cretaceous mass extinction. *Geology* **44**, 171–174 (2016).

28. S. Self, S. Blake, K. Sharma, M. Widdowson, S. Sephton, Sulfur and chlorine in late Cretaceous Deccan magmas and eruptive gas release. *Science* **319**, 1654–1657 (2008).

29. R. Choudhary, M. Santosh, B. De Vivo, G. Jadhav, E. V. S. S. K. Babu, Melt inclusion evidence for mantle heterogeneity and magma degassing in the Deccan large Igneous Province, India. *Lithos* **346-347**, 105135 (2019).

30. T. A. Mather, A. Schmidt, Environmental effects of volcanic volatile fluxes from subaerial large igneous provinces. *Large Igneous Prov. A Driv. Glob. Environ. Biot. Chang.* 103–116 (2021).

31. S. Callegaro, D. R. Baker, A. De Min, A. Marzoli, K. Geraki, H. Bertrand, C. Viti, F. Nestola, Microanalyses link sulfur from large igneous provinces and Mesozoic mass extinctions. *Geology* **42**, 895–898 (2014).

32. S. Callegaro, K. Geraki, A. Marzoli, A. De Min, V. Maneta, D. R. Baker, The quintet completed: The partitioning of sulfur between nominally volatile-free minerals and silicate melts. *Am. Mineral.* **105**, 697–707 (2020).

33. D. R. Baker, S. Callegaro, A. De Min, M. J. Whitehouse, A. Marzoli, Fluorine partitioning between quadrilateral clinopyroxenes and melt. *Am. Mineral.* **107**, 167–177 (2022).

34. Z. X. Peng, J. Mahoney, P. Hooper, C. Harris, J. Beane, A role for lower continental crust in flood basalt genesis? Isotopic and incompatible element study of the lower six formations of the western Deccan Traps. *Geochim. Cosmochim. Acta* **58**, 267–288 (1994).

35. L. Vanderkluysen, J. J. Mahoney, P. R. Hooper, H. C. Sheth, R. Ray, The feeder system of the Deccan Traps (India): Insights from dike geochemistry. *J. Petrol.* **52**, 315–343 (2011).

36. M. A. Richards, W. Alvarez, S. Self, L. Karlstrom, P. R. Renne, M. Manga, C. J. Sprain, J. Smit, L. Vanderkluysen, S. A. Gibson, Triggering of the largest Deccan eruptions by the Chicxulub impact. *Bull. Geol. Soc. Am.* **127**, 1507–1520 (2015).

37. V. S. Kale, G. Dole, P. Shandilya, K. Pande, Stratigraphy and correlations in Deccan Volcanic Province, India: Quo vadis? *Bull. Geol. Soc. Am.* **132**, 588–607 (2020).

38. L. Melluso, J. J. Mahoney, L. Dallai, Mantle sources and crustal input as recorded in high-Mg Deccan Traps basalts of Gujarat (India). *Lithos* **89**, 259–274 (2006).

39. H. C. Sheth, L. Melluso, The Mount Pavagadh volcanic suite, Deccan Traps: Geochemical stratigraphy and magmatic evolution. *J. Asian Earth Sci.* **32**, 5–21 (2008).

40. R. R. Keays, P. C. Lightfoot, Crustal sulfur is required to form magmatic Ni-Cu sulfide deposits: Evidence from chalcophile element signatures of Siberian and Deccan Trap basalts. *Miner. Depos.* **45**, 241–257 (2010).

41. B. Schoene, M. P. Eddy, C. B. Keller, K. M. Samperton. An evaluation of Deccan Traps eruption rates using geochronologic data. *Geochronology* **3**, 181–198 (2021).

42. A. E. Jay, "Volcanic architecture of the Deccan Traps, western Maharashtra, India: An integrated chemostratigraphic and paleomagnetic study," thesis, The Open University (2005).

43. L. Melluso, S. Sethna, Mineral compositions in the Deccan igneous rocks of India: An overview, in *Topics in Igneous Petrology: A Tribute to Prof. Mihir K. Bose*, J. Ray, G. Sen, B. Ghosh, Eds. (Springer, 2011), pp. 135–159.

44. A. Marzoli, P. R. Renne, R. Andreasen, R. Spiess, M. Chiaradia, D. C. S. Ruth, A. J. Tholt, K. Pande, F. Costa, The shallow magmatic plumbing system of the Deccan Traps, evidence from plagioclase megacrysts and their host lavas. *J. Petrol.* **63**, egac075 (2022).

45. A. R. Basu, A. Saha-Yannopoulos, P. Chakrabarty, A precise geochemical volcano-stratigraphy of the Deccan Traps. *Lithos* **376**, 105754 (2020).

46. M. A. Fortin, J. Riddle, Y. Desjardins-Langlais, D. R. Baker, The effect of water on the sulfur concentration at sulfide saturation (SCSS) in natural melts. *Geochim. Cosmochim. Acta* **160**, 100–116 (2015).

47. S. Beyer, M. Klemme, A. Wiedenbeck, A. Stracke, C. Vollmer, Fluorine in nominally fluorine-free mantle minerals: Experimental partitioning of F between olivine, orthopyroxene and silicate melts with implications for magmatic processes. *Earth Planet. Sci. Lett.* **337–338**, 1–9 (2012).

48. T. Thordarson, S. Self, Sulfur, chlorine and fluorine degassing and atmospheric loading by the Roza eruption, Columbia River Basalt Group, Washington, USA. *J. Volcanol. Geotherm. Res.* **74**, 49–73 (1996).

49. T. Thordarson, S. Self, N. Óskarsson, T. Hulsebosch, Sulfur, chlorine, and fluorine degassing and atmospheric loading by the 1783-1784 AD Laki (Skaftár Fires) eruption in Iceland. *Bull. Volcanol.* **58**, 205–225 (1996).

50. B. A. Black, L. T. Elkins-Tanton, M. C. Rowe, I. U. Peate, Magnitude and consequences of volatile release from the Siberian Traps. *Earth Planet. Sci. Lett.* **317-318**, 363–373 (2012).

51. P. J. Wallace, T. Plank, M. Edmonds, E. H. Hauri, *Volatiles in Magmas* (Elsevier, ed. 2, 2015), pp. 163–183.

52. J. Boulliung, B. J. Wood, SO_2 solubility and degassing behavior in silicate melts. *Geochim. Cosmochim. Acta* **336**, 150–164 (2022).

53. S. Ding, T. Plank, P. J. Wallace, D. J. Rasmussen, Sulfur_X: A model of sulfur degassing during magma ascent. *Geophys. Geosyst.* **24**, e2022GC010552 (2023).

54. Y. Liu, N. T. Samaha, D. R. Baker, Sulfur concentration at sulfide saturation (SCSS) in magmatic silicate melts. *Geochim. Cosmochim. Acta* **71**, 1783–1799 (2007).

55. C. Yallup, M. Edmonds, A. V. Turchyn, Sulfur degassing due to contact metamorphism during flood basalt eruptions. *Geochim. Cosmochim. Acta* **120**, 263–279 (2013).

56. F. M. Deegan, S. Callegaro, H. H. Svensen, C. Yakymchuk, L. E. Aradi, C. Freda, Magma – Shale interaction in large igneous provinces. *J. Petrol.* **63**, 1–10 (2022).

57. A. Lecomte, M. Cathelineau, R. Michels, C. Peiffert, M. Brouand, Uranium mineralization in the Alum

Shale Formation (Sweden): Evolution of a U-rich marine black shale from sedimentation to metamorphism. **88**, 71–98 (2017).

58. D. M. Pyle, T. A. Mather, Halogens in igneous processes and their fluxes to the atmosphere and oceans from volcanic activity: A review. *Chem. Geol.* **263**, 110–121 (2009).

59. M. R. Carroll, J. D. Webster, Solubilities of sulfur, noble gases, nitrogen, chlorine, and fluorine in magmas. *Rev. Mineral. Geochem.* **30**, 231–279 (1994).

60. T. Thordarson, S. Self, Atmospheric and environmental effects of the 1783–1784 Laki eruption: A review and reassessment. **108**, AAC 7-1–AAC 7-29 (2003).

61. K. Shimizu, M. Ito, Q. Chang, T. Miyazaki, K. Ueki, C. Toyama, R. Senda, B. S. Vaglarov, T. Ishikawa, J. I. Kimura, Identifying volatile mantle trend with the water–Fluorine–Cerium systematics of basaltic glass. *Chem. Geol.* **522**, 283–294 (2019).

62. S. Minissale, M. Casalini, C. Cucciniello, C. Balagizi, D. Tedesco, G. Boudoire, V. Morra, L. Melluso, The geochemistry of recent Nyamulagira and Nyiragongo potassic lavas, Virunga Volcanic Province, and implications on the enrichment processes in the mantle lithosphere of the Tanzania-Congo craton. *Lithos* **420-421**, 106696 (2022).

63. S. A. Gibson, E. E. Rooks, J. A. Day, C. M. Petrone, P. T. Leat, The role of sub-continental mantle as both “sink” and “source” in deep Earth volatile cycles. *Geochim. Cosmochim. Acta* **275**, 140–162 (2020).

64. S. A. Gibson, D. McKenzie, On the role of Earth’s lithospheric mantle in global volatile cycles. *Earth Planet. Sci. Lett.* **602**, 117946 (2023).

65. G. Segee-Wright, J. D. Barnes, J. C. Lassiter, D. J. Holmes, G. M. Beaudoin, R. Chatterjee, D. F. Stockli, J. E. Hoffmann, T. John, Halogen enrichment in the North American lithospheric mantle from the dehydration of the Farallon plate. *Geochim. Cosmochim. Acta* **348**, 187–205 (2023).

66. A. Aiuppa, P. Bonfanti, L. Brusca, W. D’Alessandro, C. Federico, F. Parello, Evaluation of the environmental impact of volcanic emissions from the chemistry of rainwater: Mount Etna area (Sicily).

Appl. Geochem. **16**, 985–1000 (2001).

67. M. Balagizi, M. M. Kasereka, E. Cuoco, M. Liotta, Rain-plume interactions at Nyiragongo and Nyamulagira volcanoes and associated rainwater hazards, East Africa. *Appl. Geochem.* **81**, 76–89 (2017).

68. R. Fuge, Fluorine in the environment, a review of its sources and geochemistry. *Appl. Geochem.* **100**, 393–406 (2019).

69. T. S. Tobin, P. D. Ward, E. J. Steig, E. B. Olivero, I. A. Hilburn, R. N. Mitchell, M. R. Diamond, T. D. Raub, J. L. Kirschvink, Extinction patterns, δ^{18} O trends, and magnetostratigraphy from a southern high-latitude Cretaceous-Paleogene section: Links with Deccan volcanism. *Palaeogeogr. Palaeoclimatol. Palaeoecol.* **350-352**, 180–188 (2012).

70. L. M. E. Percival, H. C. Jenkyns, T. A. Mather, A. J. Dickson, S. J. Batenburg, M. Ruhl, S. P. Hesselbo, R. Barclay, I. Jarvis, S. A. Robinson, L. Woelders, Does large igneous province volcanism always perturb the mercury cycle? Comparing the records of Oceanic Anoxic Event 2 and the end-cretaceous to other Mesozoic events. *Am. J. Sci.* **318**, 799–860 (2018).

71. F. Kaminski, A. L. Chenet, C. Jaupart, V. Courtillot, Rise of volcanic plumes to the stratosphere aided by penetrative convection above large lava flows. *Earth Planet. Sci. Lett.* **301**, 171–178 (2011).

72. L. S. Glaze, S. Self, A. Schmidt, S. J. Hunter, Assessing eruption column height in ancient flood basalt eruptions. *Earth Planet. Sci. Lett.* **457**, 263–270 (2017).

73. B. A. Black, R. R. Neely, J. F. Lamarque, L. T. Elkins-Tanton, J. T. Kiehl, C. A. Shields, M. J. Mills, C. Bardeen, Systemic swings in end-Permian climate from Siberian Traps carbon and sulfur outgassing. *Nat. Geosci.* **11**, 949–954 (2018).

74. P. Wilf, K. R. Johnson, B. T. Huber, Correlated terrestrial and marine evidence for global climate changes before mass extinction at the Cretaceous–Paleogene Boundary. *Proc. Natl. Acad. Sci. U.S.A.* **100**, 599–604 (2003).

75. J. P. Pinto, R. P. Turco, O. B. Toon, Self-limiting physical and chemical effects in volcanic eruption

clouds. *J. Geophys. Res. Atmos.* **94**, 165–174 (1989).

76. A. Robock, Volcanic eruptions and climate. *Rev. Geophys.* **38**, 191–219 (2000).

77. M. R. Rampino, S. Self, R. B. Stothers, Volcanic winters. *Annu. Rev. Earth Planet. Sci.* **16**, 73–99 (1988).

78. A. L. Chenet, V. Courtillot, F. Fluteau, M. Gérard, X. Quidelleur, S. F. R. Khadri, K. V. Subbarao, T. Thordarson, Determination of rapid Deccan eruptions across the Cretaceous-Tertiary boundary using paleomagnetic secular variation: 2. Constraints from analysis of eight new sections and synthesis for a 3500-m-thick composite section. *J. Geophys. Res. Solid Earth* **114**, B06103 (2009).

79. J. F. W. Mosselmans, P. D. Quinn, A. J. Dent, S. A. Cavill, S. D. Moreno, A. Peach, P. J. Leicester, S. J. Keylock, S. R. Gregory, K. D. Atkinson, J. R. Rosell, I18–The microfocus spectroscopy beamline at the Diamond Light Source. *J. Synchr. Rad.* **16**, 818–824 (2009).

80. V. A. Solé, E. Papillon, M. Cotte, P. Walter, J. Susini, A multiplatform code for the analysis of energy-dispersive X-ray fluorescence spectra. *Spectrochim. Acta B* **62**, 63–68 (2007).

81. J. Goldstein, D. E. Newbury, D. C. Joy, C. E. Lyman, P. Echlin, E. Lifshin, L. Sawyer, J. R. Michael, *Scanning Electron Microscopy and X-Ray Microanalysis*, (Springer, ed. 3, 2003), p. 690.

82. R. M. Rousseau, Detection limit and estimate of uncertainty of analytical XRF results. *Rigaku J.* **18**, 33–47 (2001).

83. K. P. Jochum, B. Stoll, K. Herwig, M. Willbold, A. W. Hofmann, M. Amini, S. Aarburg, W. Abouchami, E. Hellebrand, B. Mocek, I. Raczek, A. Stracke, O. Alard, C. Bouman, S. Becker, M. Dücking, H. Brätz, R. Klemd, D. de Bruin, D. Canil, D. Cornell, C. de Hoog, C. Dalpé, L. Danyushevsky, A. Eisenhauer, Y. Gao, J. E. Snow, N. Groschopf, D. Günther, C. Latkoczy, M. Guillong, E. H. Hauri, H. E. Höfer, Y. Lahaye, K. Horz, D. E. Jacob, S. A. Kasemann, A. J. R. Kent, T. Ludwig, T. Zack, P. R. D. Mason, A. Meixner, M. Rosner, K. Misawa, B. P. Nash, J. Pfänder, W. R. Premo, W. D. Sun, M. Tiepolo, R. Vannucci, T. Vennemann, D. Wayne, J. D. Woodhead, MPI-DING reference glasses for in situ microanalysis: New reference values for element concentrations and isotope ratios. *Geochem. Geophys. Geosyst.* **7**, Q02008 (2006).

84. T. Kusebauch, T. John, M. J. Whitehouse, S. Klemme, A. Putnis, Distribution of halogens between fluid and apatite during fluid-mediated replacement processes. *Geochim. Cosmochim. Acta* **170**, 225–246 (2015).

85. B. M. Urann, V. Le Roux, K. Hammond, H. R. Marschall, C. T. A. Lee, B. D. Monteleone, Fluorine and chlorine in mantle minerals and the halogen budget of the Earth's mantle. *Contrib. Mineral. Petrol.* **172**, 1–17 (2017).

86. S. Callegaro, A. Marzoli, H. Bertrand, M. Chiaradia, L. Reisberg, C. Meyzen, G. Bellieni, R. E. Weems, R. Merle, Upper and lower crust recycling in the source of CAMP basaltic dykes from southeastern North America. *Earth Planet. Sci. Lett.* **376**, 186–199 (2013).

87. J. Dinarès-Turell, T. Westerhold, V. Pujalte, U. Röhl, D. Kroon, Astronomical calibration of the Danian stage (Early Paleocene) revisited: Settling chronologies of sedimentary records across the Atlantic and Pacific Oceans. *Earth Planet. Sci. Lett.* **405**, 119–131 (2014).

88. P. J. Jugo, R. W. Luth, J. P. Richards, An experimental study of the sulfur content in basaltic melts saturated with immiscible sulfide or sulfate liquids at 1300°C and 1.0 GPa. *J. Petrol.* **46**, 783–798 (2005).

89. P. J. Jugo, M. Wilke, R. E. Botcharnikov, Sulfur K-edge XANES analysis of natural and synthetic basaltic glasses: Implications for S speciation and S content as function of oxygen fugacity. *Geochim. Cosmochim. Acta* **74**, 5926–5938 (2010).

90. T. H. Green, E. B. Watson, Crystallization of apatite in natural magmas under high pressure, hydrous conditions, with Particular Reference to 'Orogenic' Rock Series. *Contrib. Mineral. Petrol.* **79**, 96–105 (1982).

91. T. M. Harrison, E. B. Watson, The behavior of apatite during crustal anatexis: Equilibrium and kinetic considerations. *Geochim. Cosmochim. Acta* **48**, 1467–1477 (1984).

92. R. W. Le Maitre, *Igneous Rocks: A Classification and Glossary of Terms* (Cambridge Univ. Press, 2002).

93. N. Morimoto, Nomenclature of pyroxenes. *Mineral. J.* **14**, 198–221 (1989).

94. J. H. Bédard, Parameterizations of calcic clinopyroxene–Melt trace element partition coefficients. *Geochem. Geophys. Geosyst.* **15**, 303–336 (2014).

95. D. A. Neave, K. D Putirka, A new clinopyroxene-liquid barometer, and implications for magma storage pressures under Icelandic rift zones. *Am. Mineral.* **102**, 777–794 (2017).

96. T. Sano, T. Fujii, S. S. Deshmukh, T. Fukuoka, S. Aramaki, Differentiation processes of Deccan Trap basalts: Contribution from geochemistry and experimental petrology. *J. Petrol.* **42**, 2175–2195 (2001).

97. L. Melluso, M. Barbieri, L. Beccaluva, Chemical evolution, petrogenesis, and regional chemical correlations of the flood basalt sequence in the central Deccan Traps, India. *Proc. Indian Acad. Sci. (Earth Planet. Sci.)* **113**, 587–603 (2004).

98. P. A. Hoyer, K. M. Haase, M. Regelous, F. Fluteau, Systematic and temporal geochemical changes in the upper Deccan lavas: Implications for the magma plumbing system of flood basalt provinces. *Geochem. Geophys. Geosyst.* **24**, e2022GC010750 (2023).

99. M. B. Laxman, K. Vijaya Kumar, Geochemical evidences for possible absence of cu-sulfide deposits in the Deccan Volcanic Province, India. *J. Geol. Soc. India* **92**, 393–403 (2018).
